# SnRK2 kinases sense molecular crowding and form condensates to disrupt ABI1 inhibition

**DOI:** 10.1126/sciadv.adr8250

**Published:** 2025-01-29

**Authors:** Xian-Ping Yuan, Yang Zhao

**Affiliations:** ^1^ Key Laboratory of Plant Carbon Capture, Shanghai Center for Plant Stress Biology, CAS Center for Excellence in Molecular Plant Sciences, Chinese Academy of Sciences, Shanghai 200032, China.; ^2^ University of Chinese Academy of Sciences, Beijing 100049, China.

## Abstract

Plants sense and respond to hyperosmotic stress via quick activation of sucrose nonfermenting 1–related protein kinase 2 (SnRK2). Under unstressed conditions, the protein phosphatase type 2C (PP2C) in clade A interact with and inhibit SnRK2s in subgroup III, which are released from the PP2C inhibition via pyrabactin resistance 1–like (PYL) abscisic acid receptors. However, how SnRK2s are released under osmotic stress is unclear. Here, we outline how subgroup I SnRK2s sense molecular crowding to interrupt PP2C-mediated inhibition in plants. Severe hyperosmotic stress triggers condensate formation to activate the subgroup I SnRK2s, which requires their intrinsically disordered region. PP2Cs interact with and inhibit subgroup I SnRK2s, and this interaction is disrupted by phase separation of SnRK2s. The subgroup I SnRK2s are critical for severe osmotic stress responses. Our findings elucidate a mechanism for how macromolecular crowding is sensed in plants and demonstrate that physical separation of signaling molecules can segregate negative regulators to initiate signaling.

## Teaser

Subgroup I SnRK2 kinases form condensates to interrupt PP2C phosphatase–mediated inhibition under severe osmotic stress in plants.

## INTRODUCTION

**Fig. 1.**
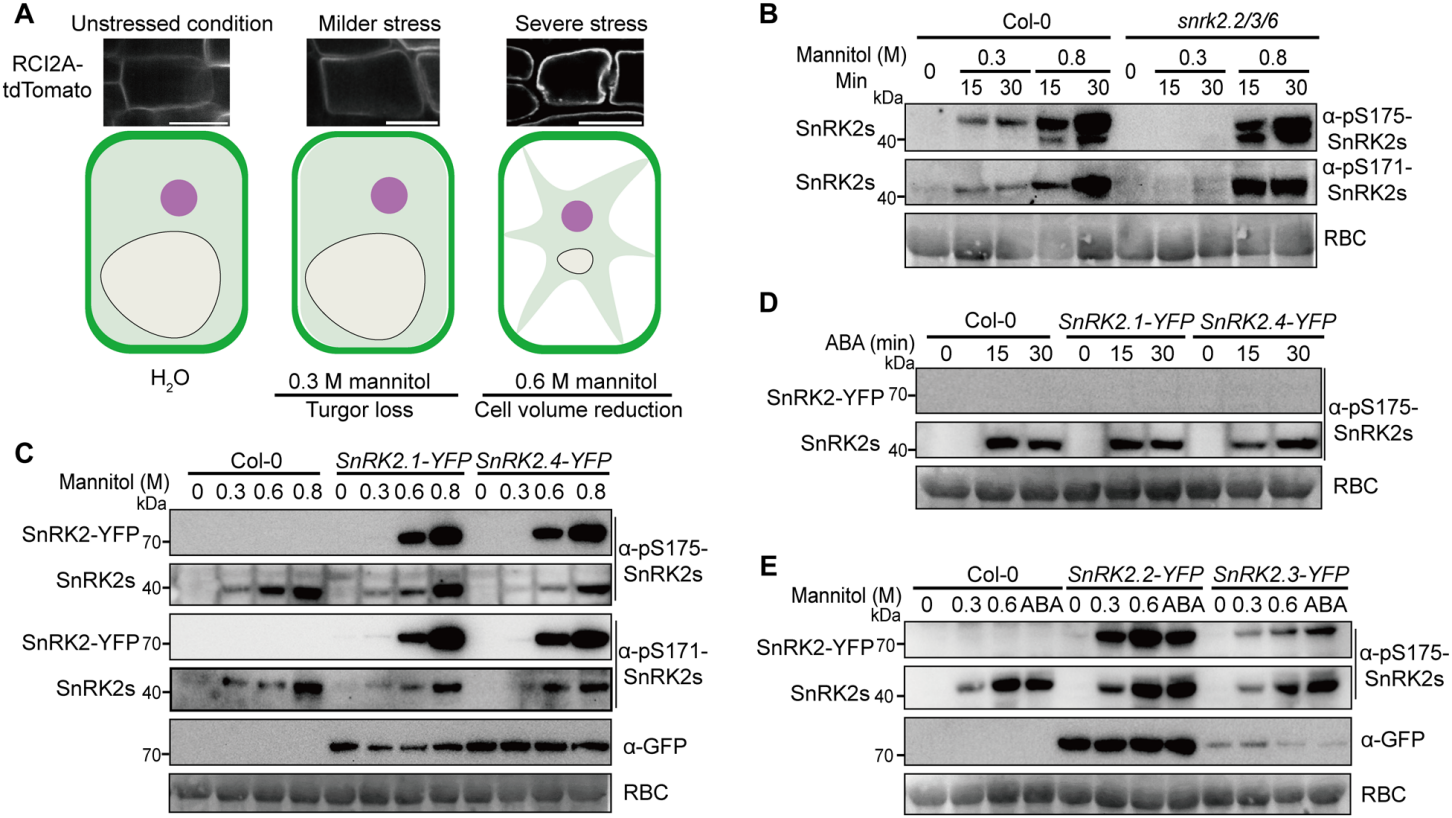
Differential activation of SnRK2s from subgroups III and I under milder and severe osmotic stresses. (**A**) Milder osmotic stress with an isoosmotic solution (0.3 M mannitol) triggers turgor loss in plants, which allows membranes to pull slightly away from the cell wall. In contrast, severe osmotic stress (e.g., 0.6 M mannitol) reduces cell fluid volume. The cellular responses of root columella cells to 10-min treatments with 0.3 or 0.6 M mannitol were monitored using the plasma membrane marker RCI2A-tdTomato. (**B**) Phosphorylation of SnRK2s in 7-day-old WT or *snrk2.2/3/6* mutant plants after treatments with 0.3 or 0.8 M mannitol for indicated time points. (**C**) Phosphorylation of YFP-tagged SnRK2.1 and SnRK2.4 (about 70 kDa) and endogenous SnRK2s (about 40 kDa) after 30-min treatments with different concentrations of mannitol (0.3, 0.6, and 0.8 M) in 7-day-old WT or *SnRK2-YFP* overexpression lines driven by the *35S* promoter. (**D**) Phosphorylation of YFP-tagged SnRK2.1 and SnRK2.4 (about 70 kDa) and endogenous SnRK2s (about 40 kDa) after treatment with 50 μM ABA for 0 to 30 min in 7-day-old WT or *SnRK2-YFP* overexpression lines. (**E**) Phosphorylation of YFP-tagged SnRK2.2 and SnRK2.3 (about 70 kDa) and endogenous SnRK2s (about 40 kDa) after 30-min treatments with different concentrations of mannitol (0.3 and 0.6 M) or 50 μM ABA in 7-day-old WT or *SnRK2-YFP* overexpression lines driven by the *35S* promoter. SnRK2 phosphorylation was detected with anti-phospho-S175-SnRK2s (B to E) and anti-phospho-S171-SnRK2s antibodies (B and C). Protein loading was detected by an anti-GFP antibody (C and E) and Ponceau S staining for Rubisco (RBC) (B to E). All experiments were repeated at least three times with similar results.

Hyperosmotic stress caused by drought and salinity threatens plant growth and survival. While animal cells require isoosmotic conditions, plant cells favor hypoosmotic conditions, which maintain turgor pressure. Mild osmotic stress below or equal to the isoosmotic level triggers turgor reduction in plants, which may be sensed by plasma membrane–localized proteins. Severe osmotic stress above the isoosmotic level reduces the fluid volume in the cytosol and nucleus in both plants and animals (Fig. 1A and fig. S1A) (*1*), which triggers crowding of abundant intracellular molecules, some of which form condensates and behave like phase-separated liquids. The transcriptional regulator SEUSS senses nuclear molecular crowding via phase separation and contributes to stress-responsive gene expression (*2*). In animal cells, the with-no-lysine kinases (WNKs) sense macromolecular crowding (via cytosolic condensates) and initiate a phosphorylation cascade to restore cell volume under hyperosmotic stress (*3*). Despite extensive studies on osmotic stress signaling, it is still unclear how core signaling components sense and respond to macromolecular crowding in plants. Kinases in the conserved sucrose nonfermenting 1–related protein kinase 2 (SnRK2) family are central positive regulators of osmotic signaling (*4*, *5*). Some members of subgroup I SnRK2s localize to punctate structures or processing bodies (P-bodies), which are cytosolic membrane-free condensates that contain nontranslating mRNAs and mRNA decapping–related proteins and contribute to stress responses (*6*–*8*). Subgroup I SnRK2s can be activated in protoplasts that lack cell walls and turgor by hyperosmotic stress (*6*, *9*), raising the question of whether and how SnRK2s respond to macromolecular crowding.

SnRK2s are repressed under unstressed conditions to prevent activation of stress responses. Under stress, SnRK2s are released from inhibition and subsequently activated by Raf-like protein kinases (RAFs) in subgroup B (*10*–*14*). The subgroup III SnRK2s, including SnRK2.2/2.3/2.6 (fig. S1B), are repressed by the clade A phosphatase type 2C (PP2C), and this inhibition is released when abscisic acid (ABA) binds members in the pyrabactin resistance 1/pyrabactin resistance 1–like/regulatory components of the ABA receptor (PYR/PYL/RCAR) family (*15*–*18*). Under osmotic stress, the receptor-like cytoplasmic kinase Botrytis-induced kinase 1 (BIK1) releases SnRK2.6 from PP2C binding and inhibition via tyrosine phosphorylation of SnRK2.6 (*19*). The subgroup I member SnRK2.4 is strongly repressed by the clade A PP2C ABA-insensitive 1 (ABI1) and weakly repressed by PP2CA (*20*–*22*). However, it is unclear how the other four SnRK2s in subgroup I are repressed. Since SnRK2 activation by osmotic stress is not defective in the *pyl* duodecuple mutant (*23*), the osmotic stress–mediated release of subgroup I SnRK2s should be a unique process that differs from PYL-mediated disinhibition of subgroup III SnRK2s. The idea that subgroup I SnRK2s are released via a unique mechanism is also supported by their distinct localization in P-bodies (*6*), their distinct activation by cell volume reduction but not ABA in protoplasts (*9*), and their distinct function in salt and osmotic stress responses but not ABA responses (*4*, *6*, *24*). In addition, members of the B4 RAFs that activate subgroup I SnRK2s also localize to P-bodies (*12*, *13*). However, it remains unclear whether the specific localization of subgroup I SnRK2s is involved in their disinhibition and activation under osmotic stress.

In our research, we found that subgroup I SnRK2s, including SnRK2.1/2.4/2.5/2.9/2.10 (fig. S1B), sense molecular crowding to form cytosolic condensates, which leads to the spatial segregation of SnRK2s from their negative regulators, the PP2Cs, thereby releasing subgroup I SnRK2s from inhibition to activate stress responses. The subgroup III SnRK2s are activated under the milder stress that triggers turgor reduction, while subgroup I SnRK2s are activated under the severe stress that leads to molecular crowding. Both condensate formation and activation of subgroup I SnRK2s require their intrinsically disordered region (IDR) domains. Our findings elucidate how plants respond to molecular crowding under severe osmotic stress and uncover a unique mechanism for releasing signaling molecules from negative regulators.

## RESULTS

### Differential activation of SnRK2s from subgroups III and I under mild and severe osmotic stresses

Osmotic stress is a physical stimulus that triggers many physiological changes that plants may sense (*1*). Since mild and severe osmotic stresses trigger distinct physiological changes at the cellular level (Fig. 1A and fig. S1A), we analyzed whether different subgroups of SnRK2s were differentially activated by varying degrees of stress. The osmolarity of *Arabidopsis* seedlings grown on ½ MS medium is about 280 to 290 mosmol/kg (corresponding to 301 to 312 mosmol/liter, fig. S1C). Therefore, we consider 300 mM mannitol treatment an isoosmotic or milder treatment. Using the anti-phospho-S175-SnRK2s and anti-phospho-S171-SnRK2s antibodies (*10*, *23*), we detected the phosphorylation of serine residues in the activation loop of multiple SnRK2s corresponding to Ser^175^ and Ser^171^ in SnRK2.6 under osmotic stress. SnRK2s are quickly activated by either milder (300 mM mannitol) or severe (800 mM mannitol) osmotic stresses (Fig. 1B). SnRK2 activation by 300 mM mannitol was undetectable in the *snrk2.2/3/6* mutant (Fig. 1B), indicating that the subgroup III (i.e., SnRK2.2/3/6) but not subgroup I SnRK2s were activated by milder stress (which triggers turgor loss). SnRK2 activation by 300 mM mannitol was also reduced in the *snrk2.1/4/5/7/8/9/10* septuple mutant but not the *snrk2.1/4/5/9/10* quintuple mutant (fig. S1D), suggesting that the subgroup II kinases SnRK2.7 and SnRK2.8 may also be activated by milder stress. In contrast, SnRK2 activation by 800 mM mannitol was comparable to the wild type (WT) in the *snrk2.2/3/6* triple mutant (Fig. 1B) but reduced in the *snrk2.1/4/5/9/10* quintuple, *snrk2.1/4/5/7/8/9/10* septuple, and *snrk2.2/3/6* mutants under 600 mM mannitol treatment (fig. S1D), suggesting that SnRK2s in subgroups I and III were activated by severe stress (which triggers turgor loss and cell volume reduction). The use of the anti-phospho-S158-SnRK2.1 antibody, which mainly recognizes phosphorylation of subgroup I SnRK2s, showed that high levels of SnRK2 activation can be observed in WT and *snrk2.2/3/6* but not in the *snrk2.1/4/5/9/10* quintuple and *snrk2.1/4/5/7/8/9/10* septuple mutants under 600 mM mannitol treatment (fig. S1D). In addition, SnRK2 activation by ABA is undetectable in the *snrk2.2/3/6* triple mutant but was comparable to WT in the *snrk2.1/4/5/7/8/9/10* septuple mutant (fig. S1E). These results suggested that the subgroup I SnRK2s are specifically activated by severe osmotic stress, and the subgroup II SnRK2s are activated by mild osmotic stress, while the subgroup III SnRK2s are activated by ABA and milder or severe osmotic stresses.

To confirm the unique activation profile of subgroup I SnRK2s, we generated transgenic lines expressing yellow fluorescent protein (YFP)–tagged fusions of five SnRK2 proteins from subgroup I that are activated by hyperosmotic stress in protoplasts (*9*), namely SnRK2.1, SnRK2.4, SnRK2.5, SnRK2.9, and SnRK2.10. Unlike protoplasts that lack cell walls and turgor, transgenic plants can be used to detect signaling triggered by the reduction of cell turgor and cell volume. The YFP-tagged SnRK2.1, SnRK2.4, SnRK2.5, and SnRK2.10 protein kinases were strongly activated by 600 and 800 mM mannitol but not ABA treatments, while ABA activated the endogenously expressed SnRK2s, as detected by the phosphorylation of Ser^175^ and Ser^171^ of SnRK2s (Fig. 1, C and D; and fig. S1, F and G). The cellular volume is decreased in a two-phase mode, a nearly linear mode before turgor loss, followed by a hyperbolic decay when turgor is absent (*25*). Therefore, the subgroup I SnRK2s could be very weakly activated by 300 mM mannitol treatment in the *SnRK2* overexpression lines (fig. S1F). Similarly, SnRK2.9-YFP was weakly activated by 600 mM mannitol but not ABA or 300 mM mannitol treatments (fig. S1H). In contrast, the SnRK2.2-YFP, SnRK2.3-YFP, and SnRK2.6-GFP protein kinases (all subgroup III proteins) were activated by ABA or 300 and 600 mM mannitol treatments (Fig. 1E and fig. S1, I and J). Together, our data demonstrated that subgroup I SnRK2s are mainly activated by severe osmotic stress, which differs from subgroup III SnRK2s that are activated by mild and severe osmotic stresses and ABA.

### Severe osmotic stress triggers the condensation of subgroup I SnRK2s

Severe osmotic stress reduces cell volume and results in macromolecule crowding, which triggers rapid and reversible formation of condensates containing multivalent proteins (*26*). The SnRK2 proteins have highly acidic C-terminal regions, and SnRK2.1 and SnRK2.4 localize to P-bodies or punctate structures under severe osmotic stress (*6*–*8*, *27*). When transiently expressed in *Nicotiana benthamiana* leaves, the punctate structures of SnRK2.1-GFP and SnRK2.4-GFP largely overlapped with the P-body marker DCP1-mCherry after 500 mM mannitol treatment (fig. S2, A and B). However, whether these proteins sense molecular crowding to form condensates and become activated remains unclear.

**Fig. 2.**
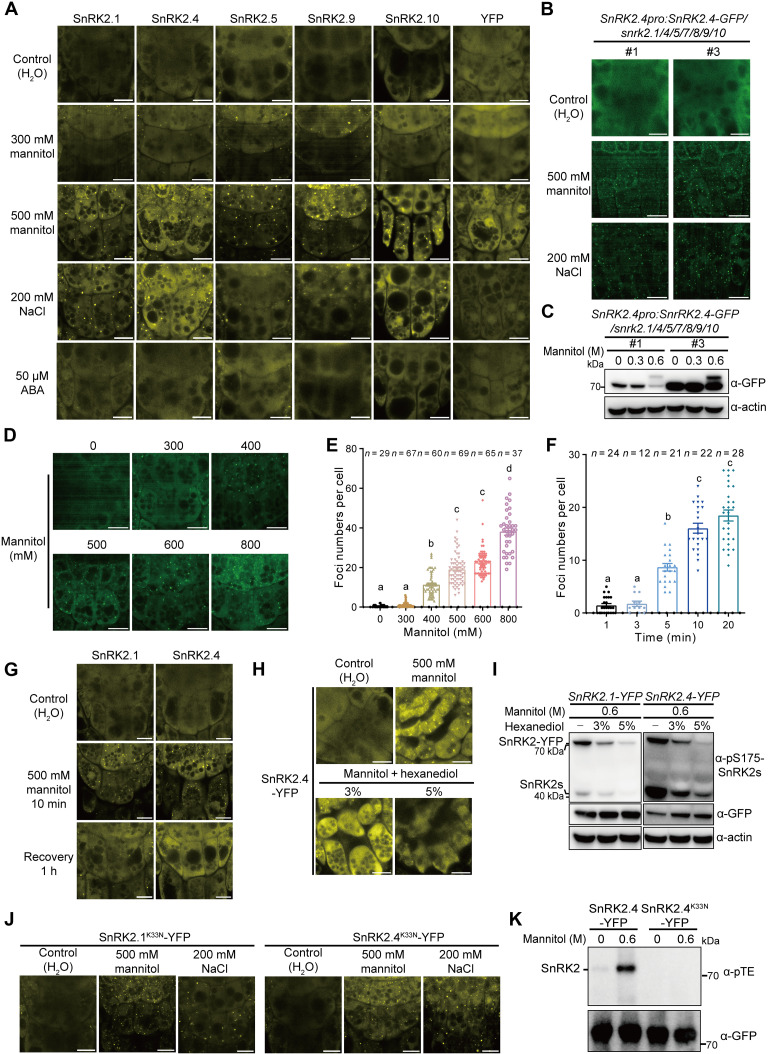
Severe osmotic stress triggers formation of punctate structures containing subgroup I SnRK2s. (**A**) Representative confocal images of YFP-tagged SnRK2.1/4/5/9 or 10 in *Arabidopsis* root tip cells of the respective *SnRK2-YFP* overexpression lines after 10-min mannitol, 20-min NaCl, or 30-min ABA treatments. (**B** to **F**) Representative confocal images of GFP-tagged SnRK2.4 (B) and SnRK2.1 (D) in root tip cells of *SnRK2pro:SnRK2-GFP* transgenic seedlings in the *snrk2.1/4/5/7/8/9/10* mutant background after 10-min mannitol or 20-min NaCl treatments. The foci numbers of SnRK2.1-GFP were quantified after 10-min mannitol treatments (E) or 500 mM mannitol treatment for indicated time points (F). Error bars indicate SEM; *n* ≥ 29 cells (E) and *n* ≥ 12 cells (F). Letters indicate significant differences according to one-way ANOVA followed by a Tukey test (E and F). Band shift of SnRK2.4-GFP in 7-day-old seedlings after 30-min mannitol treatments (C). SnRK2.4-GFP was detected by an anti-GFP antibody (top). (**G**, **H**, and **J**) Confocal images of YFP-tagged WT (G and H) or “kinase-dead” forms (J) of SnRK2.1 or SnRK2.4, in root tip cells of *SnRK2-YFP* overexpression lines, after mannitol and NaCl treatments (J), or with HEX (H), or recovery treatment with water (G). (**I**) Phosphorylation of YFP-tagged SnRK2.1 and SnRK2.4 (about 70 kDa) and endogenous SnRK2s (about 40 kDa) in 7-day-old *SnRK2-YFP* overexpression lines after mannitol treatment with HEX. SnRK2 phosphorylation was detected with the anti-phospho-S175-SnRK2s antibody. Protein loading was detected by anti-GFP and anti-actin antibodies. (**K**) Autophosphorylation activity of WT and “kinase-dead” SnRK2.4-YFP immunoprecipitated from the *SnRK2.4-YFP* overexpression lines after 30-min mannitol treatment. After the PNBM alkylation reaction, the thiophosphate ester groups on the substrate were detected by the anti-thiophosphate ester antibody (α-pTE). Scale bars in (A), (B), (D), (G), (H), and (J) were 10 μm. All experiments were repeated at least three times with similar results.

Since the subgroup I SnRK2s were mainly activated by severe osmotic stress, which triggers molecular crowding (Fig. 1 and fig. S1), we proposed that these SnRK2s may sense molecular crowding to trigger condensate formation and kinase activation. Therefore, we analyzed the condensate formation of all subgroup I SnRK2s under severe osmotic stress using transgenic lines overexpressing *SnRK2s-YFP* or with native promoter-driven *SnRK2s*. All overexpressed subgroup I SnRK2s, including SnRK2.1, SnRK2.4, SnRK2.5, SnRK2.9, and SnRK2.10, formed condensates upon treatment with 500 mM mannitol or 200 mM NaCl in root columella cells that lack the central vacuoles, while ABA treatment did not induce condensate formation (Fig. 2A). SnRK2.1-GFP and SnRK2.4-GFP, expressed using their native promoters in the *snrk2.1/4/5/7/8/9/10* septuple mutant background, also formed condensates upon 500 mM mannitol or 200 mM NaCl treatments, although smaller and more dense compared with those of the overexpressed SnRK2s, probably because of the lower oligomerization propensity of the enhanced green fluorescent protein (GFP) tag compared with the enhanced YFP tag (Fig. 2B and fig. S2C). Band shifts and activation of the GFP-tagged SnRK2.1 and SnRK2.4 protein kinases were strongly triggered by 600 mM mannitol but not 300 mM mannitol treatment, as detected using *SnRK2.1* and *SnRK2.4* transgenic lines driven by their native promoters (Fig. 2C and fig. S2, D and E). Besides, SnRK2.1 condensation occurred at 400 to 800 mM mannitol treatments but not at 300 mM mannitol treatment (Fig. 2, D and E). The density of SnRK2.1 condensates was gradually elevated with increased concentrations of mannitol and duration of treatment (Fig. 2, E and F). We further investigated whether the cytosolic or nuclear localization of SnRK2s affects condensate formation using transgenic lines overexpressing *SnRK2.1-YFP* fused with peptides containing either a nuclear localization signal or a nuclear export signal. The cytosol-localized SnRK2.1-YFP formed condensates upon 500 mM mannitol treatment, while the nuclear-localized SnRK2.1-YFP could not form punctate structures (fig. S2F). These results demonstrated that subgroup I SnRK2s respond to macromolecular crowding caused by cell volume reduction under severe osmotic stress in a concentration-dependent manner.

After condensate formation triggered by hyperosmotic solution, the condensates of SnRK2.1-YFP and SnRK2.4-YFP were dissolved by recovery in hypoosmotic solution, suggesting the reversible nature of SnRK2 condensates (Fig. 2G). Most reversible biomolecular condensates are formed through liquid-liquid phase separation (LLPS) (*28*). To investigate whether subgroup I SnRK2s undergo LLPS, we analyzed the properties of SnRK2 condensates using 1,6-hexanediol (HEX) treatment and fluorescence recovery after photobleaching (FRAP). The alcohol HEX is widely used to dissolve LLPS condensates (*29*). The SnRK2.4-YFP puncta were reduced by 3% HEX cotreatment and disappeared after 5% HEX cotreatment (Fig. 2H). Activation of SnRK2.1-YFP, SnRK2.4-YFP, and endogenous SnRK2s by osmotic stress was reduced by 3% HEX cotreatment (Fig. 2I); in contrast, activation of endogenous SnRK2s by ABA was not affected by 3% HEX cotreatment (fig. S2G). Moreover, the spatiotemporal FRAP assay showed that the SnRK2.1-GFP condensate can redistribute rapidly from the unbleached region to the bleached loci (fig. S2, H and I) and that SnRK2.1-GFP droplets can fuse to form larger droplets (fig. S2J and movie S1). These results suggest that subgroup I SnRK2s form condensates via LLPS under severe osmotic stress.

In contrast, the overexpressed SnRK2.2-YFP, SnRK2.3-YFP, SnRK2.7-YFP, and SnRK2.8-YFP proteins were unable to form punctate structures under either severe osmotic stress or ABA treatment but did exhibit enhanced nuclear signals upon severe osmotic stress (fig. S2K). To our surprise, SnRK2.6-GFP also formed condensates in the *SnRK2.6pro:SnRK2.6-GFP* transgenic lines in the *ost1* mutant background (fig. S2K), supporting the central function of SnRK2.6 in either ABA or osmotic stress responses. The HEX cotreatment reduced condensates and SnRK2 activation under osmotic stress (Fig. 2, H and I), raising a question of whether kinase activity is required for condensate formation. We therefore generated transgenic lines overexpressing the catalytically inactive variants *SnRK2.1*^*K33N*^ and *SnRK2.4*^*K33N*^. These mutations did not affect condensate formation of SnRK2.1 and 2.4 upon severe osmotic stress (Fig. 2J) but did block SnRK2 activation by 600 mM mannitol treatment in transgenic lines or during in vitro kinase assay with purified recombinant proteins (Fig. 2K and fig. S2, L and M). These results demonstrated that condensate formation does not require kinase activation of subgroup I SnRK2s but may come before kinase activation.

### The IDR is required for condensation and activation of subgroup I SnRK2s

**Fig. 3.**
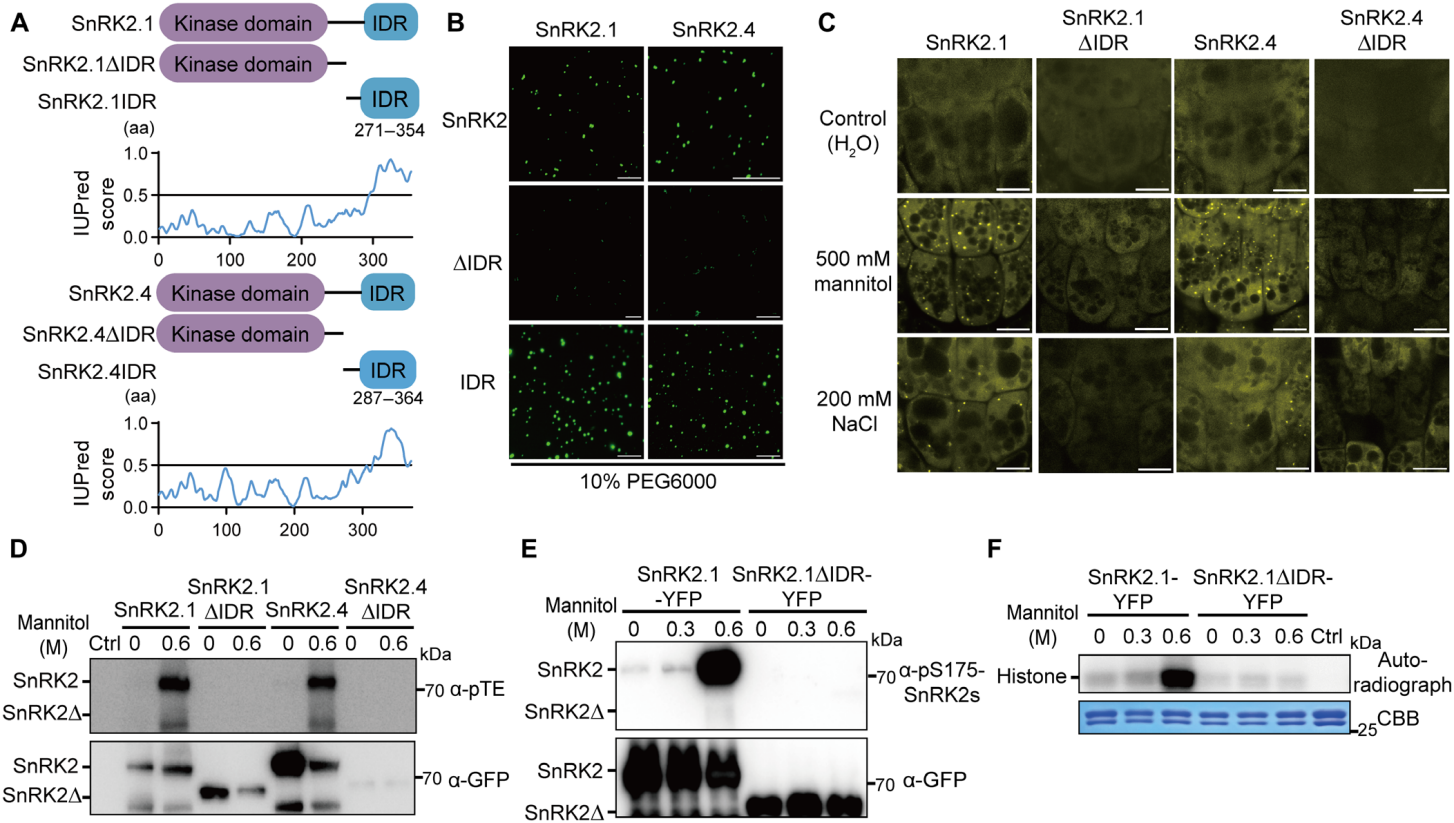
The IDR is required for condensation and activation of subgroup I SnRK2s. (**A**) Top: Protein domain structures of SnRK2.1, SnRK2.4, and their truncations. Bottom: Predictions of the disordered regions by the IUPred3 algorithm. The residue numbers of IDRs are shown. aa, amino acid. (**B**) In vitro phase separation assay of different truncations of SnRK2.1-GFP and SnRK2.4-GFP recombinant proteins in the presence of 10% PEG6000. Scale bars, 5 μm. (**C**) Representative confocal images of YFP-tagged SnRK2.1, SnRK2.4, and their truncations in *Arabidopsis* root tip cells of *SnRK2-YFP* overexpression lines driven by the *35S* promoter after 500 mM mannitol treatment for 10 min or 200 mM NaCl for 20 min. Scale bars, 10 μm. (**D**) Autophosphorylation activity of YFP-tagged SnRK2.1 and SnRK2.4 (about 70 kDa) and their truncations (about 60 kDa) immunoprecipitated from the *SnRK2-YFP* overexpression lines after 0.6 M mannitol treatment for 30 min. After the PNBM alkylation reaction, the thiophosphate ester groups on the substrate were detected by the anti-thiophosphate ester antibody (α-pTE). Loading of the SnRK2-YFP proteins was detected by an anti-GFP antibody. (**E**) Phosphorylation of YFP-tagged SnRK2.1 (about 70 kDa) and its truncation (about 60 kDa) immunoprecipitated from the respective *SnRK2-YFP* overexpression lines after 30-min treatments with different concentrations of mannitol (0, 0.3, and 0.6 M). SnRK2 phosphorylation was detected with the anti-phospho-S175-SnRK2s antibody. Protein loading was detected by an anti-GFP antibody. (**F**) Phosphorylation activity of YFP-tagged SnRK2.1 and its truncation immunoprecipitated from the *SnRK2-YFP* overexpression lines after 30-min treatments with different concentrations of mannitol (0.3 and 0.6 M). Autoradiography (top) and Coomassie staining (bottom) exhibit phosphorylation and loading of histone, respectively. All experiments except (F) (two replications) were repeated at least three times with similar results.

Most LLPS condensates are driven by IDRs within proteins (*30*). Structure prediction of SnRK2.1 and SnRK2.4 using the IUPred3 algorithm revealed that both subgroup I proteins harbor IDRs in their C-termini (Fig. 3A). To test the function of the IDRs in subgroup I SnRK2s, we purified different truncated recombinant proteins. The full-length SnRK2.1-GFP and SnRK2.4-GFP proteins formed numerous condensates after 10% polyethylene glycol 6000 (PEG6000) treatment, which triggered macromolecular crowding; however, deletion of the IDR domain markedly blocked SnRK2 condensation (Fig. 3B). The formation of SnRK2 condensates was enhanced by increased concentrations of PEG6000, suggesting that subgroup I SnRK2s are highly sensitive to the degree of molecular crowding (fig. S3, A and B). In addition, the GFP-tagged IDR domains of the two SnRK2s were sufficient to form condensates in vitro (Fig. 3B). These results suggested that the SnRK2.1 and SnRK2.4 proteins are sensitive to molecular crowding and form condensates via their IDR domains.

We further investigated IDR functions in transgenic lines overexpressing full length or truncated *SnRK2.1* and *SnRK2.4*. Deletion of the IDR domain completely blocked SnRK2 condensation in vivo upon 500 mM mannitol or 200 mM NaCl treatments (Fig. 3C). However, the YFP-tagged IDRs (IDR-YFP) of SnRK2.1/2.4 were unable to form condensates in vivo upon severe osmotic stress (fig. S3C), suggesting that the IDR domain is necessary but insufficient for phase separation of subgroup I SnRK2s in vivo.

Since kinase activity was associated with phase separation of the subgroup I SnRK2s (Fig. 2E), we further detected the kinase activity of the proteins missing the IDRs. Unexpectedly, IDR deficiency completely blocked activation of SnRK2.1 and SnRK2.4 by 600 mM mannitol in the kinase assay using immunopurified recombinant proteins (Fig. 3D and fig. S3D). Using the anti-phospho-S175-SnRK2s antibody, we confirmed that deletion of the SnRK2 IDR domain disrupts activation by severe osmotic stress (Fig. 3E and fig. S3E). The weak activation of the immunoprecipitated SnRK2.1 and SnRK2.4 under unstressed conditions could be detected occasionally when we used the immunoprecipitated SnRK2s that have higher protein levels (Fig. 3E and fig. S3D). Further, the IDR-deficiency SnRK2.1 could not phosphorylate histone after osmotic stress treatment (Fig. 3F). These results suggested that the ability to form condensates is required for the activation of subgroup I SnRK2s under severe osmotic stress.

### ABI1 interacts with and inhibits subgroup I SnRK2s

**Fig. 4.**
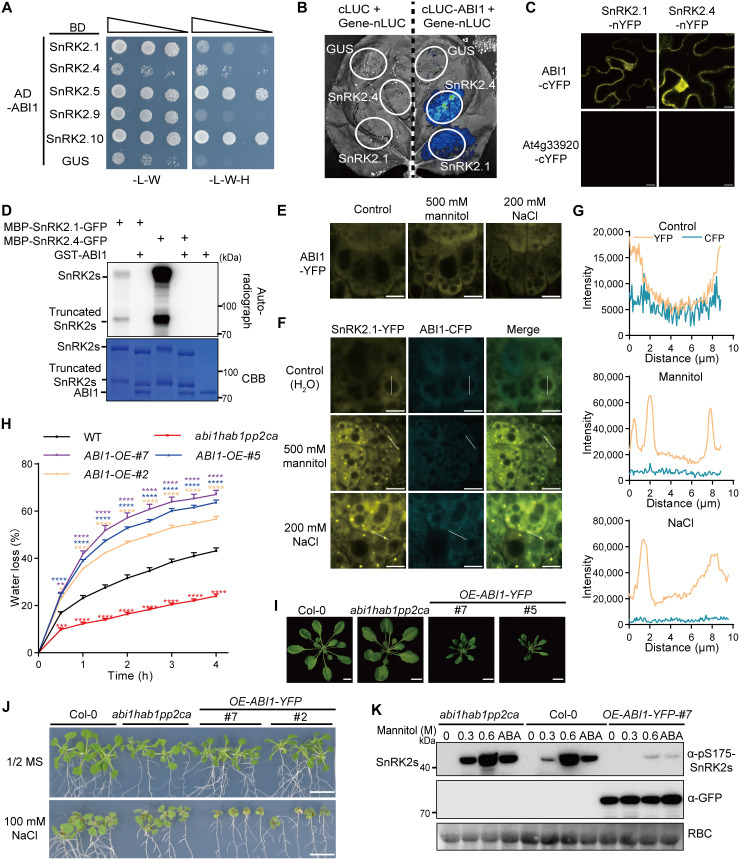
Spatial segregation via condensation releases SnRK2 kinases from ABI1. (**A**) Interactions between ABI1 and subgroup I SnRK2s in yeast two-hybrid assay. Interactions were determined by yeast growth on media lacking Leu, Trp, and His (-L-W-H) after inoculation with saturated culture dilutions. (**B** and **C**) ABI1-SnRK2 interactions in *N. benthamiana* leaves using split-LUC (B) and split-YFP (C). Scale bars, 10 μm. (**D**) Dephosphorylation and inhibition of SnRK2.1 and SnRK2.4 by recombinant GST-ABI1. Autoradiography (top) and Coomassie staining (bottom) exhibit phosphorylation and loading of proteins, respectively. (**E** and **F**) Confocal images of *Arabidopsis* root tip cells overexpressing *ABI1-YFP* (E) or co-overexpressing *SnRK2.1-YFP* and *ABI1-CFP* (F) after 10-min mannitol or 20-min NaCl treatments. Scale bars, 10 μm. (**G**) Intensity scale values of colocalization on the white lines in (F). (**H**) Cumulative transpirational water loss from rosettes of the WT (Col-0), the *abi1hab1pp2ca* triple *PP2C* mutant, and the *ABI1-YFP* overexpression lines at the indicated times after detachment. Error bars indicate SEM (*n* = 3). Asterisks indicate significant differences, two-way ANOVA followed by a Tukey test (*P < 0.05, **P < 0.01, ***P < 0.001, and ****P < 0.0001). h, hours. (**I**) Representative pictures of plant growth in the soil of the indicated lines in (H). Scale bars, 1 cm. (**J**) Shoots of the WT (Col-0) and *abi1hab1pp2ca* and *ABI1-YFP* overexpression lines 12 days after the seedlings were transferred to ½ MS medium with or without 100 mM NaCl. Scale bars, 1 cm. (**K**) Phosphorylation of endogenous SnRK2s after 30-min treatments with 0.3 and 0.6 M mannitol or 50 μM ABA in 7-day-old WT (Col-0) and *abi1hab1pp2ca* and *ABI1-YFP* overexpression lines. SnRK2 phosphorylation was detected with the anti-phospho-S175-SnRK2s antibody. Protein loading was detected by an anti-GFP antibody and Ponceau S staining. All experiments were repeated at least three times with similar results.

SnRK2s are inhibited under unstressed conditions and must be released from inhibition to become active, which is exerted by PYLs under stressed conditions in the presence of ABA (*15*–*18*). Although SnRK2.4 is repressed by ABI1 and PP2CA (*20*–*22*), it is unclear how the other four SnRK2s in subgroup I are repressed under unstressed conditions and whether other PP2Cs are their negative regulators. First, we analyzed PP2C-SnRK2 interactions using yeast two-hybrid and bimolecular fluorescence complementation (BiFC) assays. We fused the subgroup I SnRK2s to the GAL4 DNA-binding domain and ABI1 to the GAL4-activating domain. Interactions between ABI1 and SnRK2.1/2.4/2.5/2.10 were detected but not the subgroup I SnRK2.9 (Fig. 4A and fig. S4A). We confirmed the PP2C-SnRK2 interactions using split-luciferase (LUC) complementation assays in *N. benthamiana* leaves. Two clade A PP2Cs, ABI1 and HAB1, strongly interacted with SnRK2.1 and SnRK2.4, and PP2CA weakly interacted with SnRK2.1 and SnRK2.4 (Fig. 4B and fig. S4B). In addition, an ABI1-cYFP fusion reconstituted the YFP signals when expressed with the nYFP (N-fragment YFP)–tagged SnRK2.1 and SnRK2.4 in both the cytosol and nucleus in *N. benthamiana* leaves, but the clade D PP2C At4g33920 cYFP fusion did not (Fig. 4C).

Next, we investigated the potential regulation of subgroup I SnRK2s by PP2Cs using in vitro kinase assays. The kinase activities of recombinant GFP-tagged SnRK2.1 and SnRK2.4 were completely repressed by the purified recombinant GST-ABI1 (Fig. 4D). Using the anti-phospho-S175-SnRK2s antibody, we confirmed that autophosphorylation of SnRK2.1 and SnRK2.4 was repressed by ABI1 but not by the clade D PP2C At4g33920 (fig. S4C). ABI1-mediated dephosphorylation of SnRK2.1 and SnRK2.4 resulted in a band shift of the protein kinases, as detected by Coomassie brilliant blue staining or Western blot with the anti-GFP antibody (Fig. 4D and fig. S4C). These results demonstrated that members of clade A PP2Cs are also negative regulators of subgroup I SnRK2s.

### Spatial segregation via condensation releases SnRK2 kinases from ABI1

Since condensate formation was essential for kinase activation (Figs. 2I and 3, D to F), we proposed that condensation may contribute to the release or subsequent activation of subgroup I SnRK2s. First, we investigated whether the clade A PP2Cs form condensates under severe osmotic stress. Using transgenic lines overexpressing PP2Cs-YFP, we detected nuclear localization of PP2CA and cytosolic localization of ABI1 and HAB1, but YFP-tagged ABI1, HAB1, and PP2CA were unable to form punctate structures under severe osmotic stress (Fig. 4E and fig. S4D). Second, we analyzed whether condensation separates SnRK2 from ABI1 in transgenic lines overexpressing combinations of *SnRK2.1-YFP* and *ABI1-CFP* or *SnRK2.1-GFP* and *mRFP-ABI1*. SnRK2.1-YFP or SnRK2.1-GFP fusions formed condensates upon 500 mM mannitol or 200 mM NaCl treatments, but neither ABI1-CFP nor mRFP-ABI1 generated punctate structures (Fig. 4F and fig. S4E). Therefore, condensate formation spatially separated SnRK2.1 from ABI1 (Fig. 4G and fig. S4F). SnRK2 activation analysis of those overexpression lines indicated that lines coexpressing *SnRK2.1-YFP* and *ABI1-CFP* showed similar activation levels as lines overexpressing just *SnRK2.1-YFP* (fig. S4G). Last, we investigated whether depletion of PP2Cs affects SnRK2 condensation in transgenic lines overexpressing *SnRK2-YFP* in the *abi1hab1pp2ca* triple mutant background. SnRK2.1-YFP and SnRK2.4-YFP proteins still formed condensates upon severe osmotic stress but not under unstressed conditions without the three clade A PP2Cs (fig. S4H). These results suggested that spatial segregation via condensation releases subgroup I SnRK2s from PP2Cs upon severe osmotic stress.

We further investigated the biological significance of clade A PP2Cs in SnRK2 activation and osmotic stress responses. Overexpression of *ABI1* resulted in accelerated water loss in detached rosettes and defective plant growth under 100 mM NaCl, while the *abi1hab1pp2ca* mutant exhibited reduced water loss in detached rosettes (Fig. 4, H to J), supporting the negative roles of PP2Cs in osmotic stress responses. Consistent with the negative roles of PP2Cs in ABA signaling, the *ABI1* overexpression lines were less sensitive to ABA, while the *abi1hab1pp2ca* mutant was hypersensitive to ABA (fig. S5A). SnRK2 activation by 300 and 600 mM mannitol or ABA was markedly reduced in the *ABI1* overexpression lines while enhanced in the *abi1hab1pp2ca* mutant (Fig. 4K and fig. S5B). Likewise, SnRK2 activation in the *PP2CA* and *HAB1* overexpression lines was slightly reduced (fig. S5, C and D). These results indicated that clade A PP2Cs are broad negative regulators of SnRK2s in subgroups I and III.

We further analyzed interactions between ABI1 and the IDR domain or the IDR-deleted truncated SnRK2 proteins. The IDR domains, but not the IDR-deleted truncated SnRK2 proteins, have a strong interaction with ABI1 in the yeast two-hybrid and BiFC assays (fig. S5, E and F), suggesting that ABI1 may not inhibit these IDR-deleted proteins in vivo. However, we cannot rule out the possibility that other negative regulators repress the subgroup I SnRK2s.

### Subgroup I SnRK2s control osmotic stress resistance

**Fig. 5.**
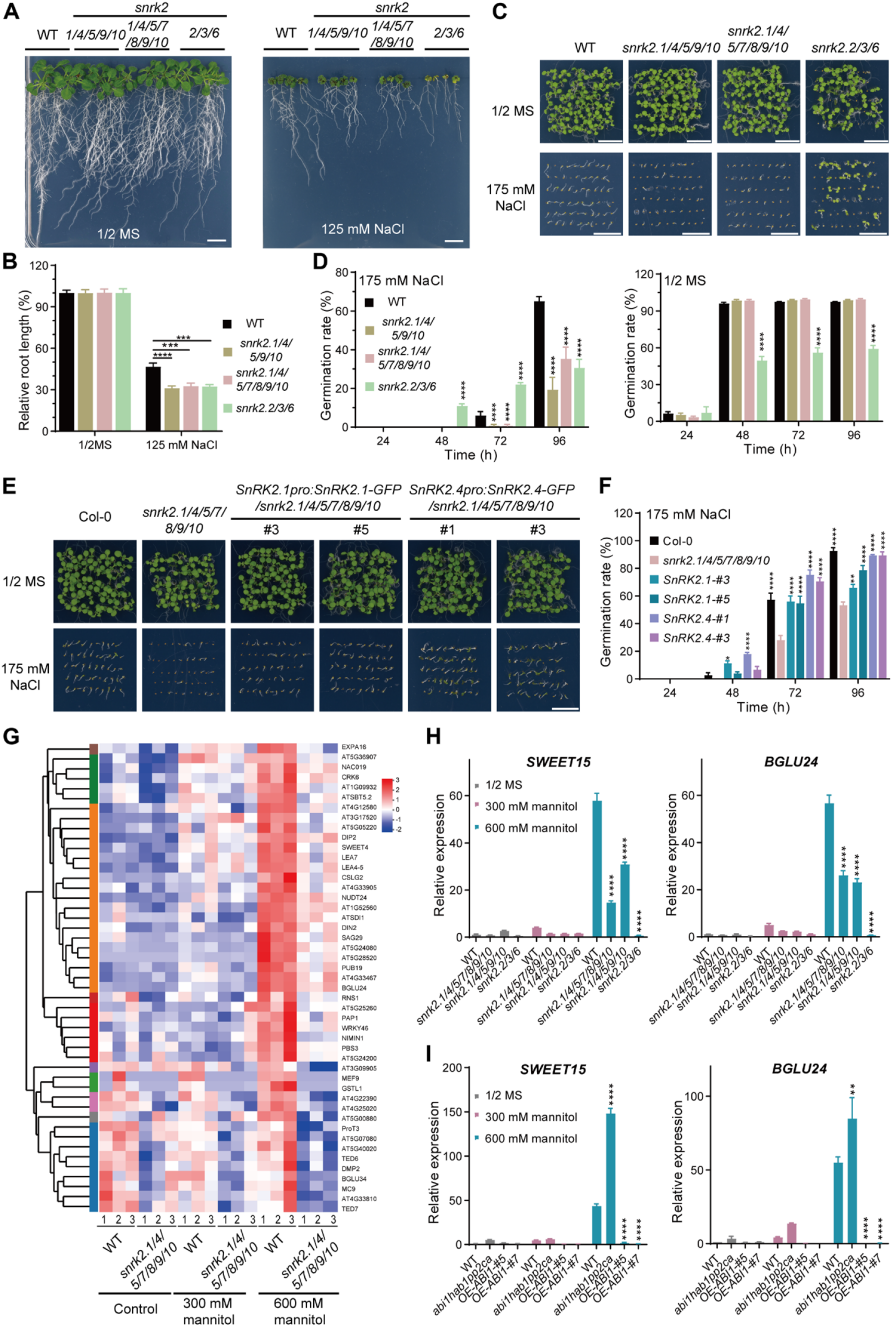
Subgroup I SnRK2s control osmotic stress resistance. (**A** and **B**) Seedling growth of WT (Col-0) and the *snrk2* higher-order mutants, namely *snrk2.2/3/6*, *snrk2.1/4/5/9/10*, and *snrk2.1/4/5/7/8/9/10*, 12 days after the seedlings were transferred to ½ MS medium with or without 125 mM NaCl. Root length was quantified (B). Values are means ± SEM (*n* = 11 independent seedlings). Scale bars, 1 cm. (**C** and **D**) Seed germination of WT and *snrk2* mutants on ½ MS medium with or without 175 mM NaCl. Scale bars, 1 cm. Seed germination rate was quantified (D). h, hours. Values are means ± SEM. (**E** and **F**) Seed germination of WT, *snrk2.1/4/5/7/8/9/10* mutant, and *SnRK2pro:SnRK2-GFP* transgenic lines expressing *SnRK2.1-GFP* and *SnRK2.4-GFP* in the *snrk2.1/4/5/7/8/9/10* mutant background on ½ MS medium with or without 175 mM NaCl. Scale bars, 1 cm. The seed germination rate was quantified (F). (**G**) Heatmaps showing the relative expression changes of genes responsive to osmotic stress after treatments with 300 or 600 mM mannitol for 6 hours with three replicates. (**H** and **I**) Relative expression of selected osmotic stress–responsive genes in WT, *snrk2* mutants (H), and the *abi1hab1pp2ca* triple mutant and *ABI1-YFP* overexpression lines (I), as determined by quantitative RT-PCR. (B, D, F, H, and I) Error bars represent SEM (*n* = 3 biological independent replicates). Asterisks indicate significant differences, two-way ANOVA followed by a Tukey test (*P < 0.05, **P < 0.01, ***P < 0.001, and ****P < 0.0001). All experiments except (G) (conducted once in triplicate) were repeated at least three times with similar results.

Since subgroup I SnRK2s were mainly activated under severe but not milder osmotic stress or ABA treatment (Fig. 1 and fig. S1), we further investigated the biological significance of subgroup I SnRK2s in osmotic stress responses. We first evaluated seed germination and seedling growth under osmotic stress using mutants of different subgroups of *SnRK2*s, including the *snrk2.2/3/6* triple mutant that lacks all subgroup III *SnRK2*s, the *snrk2.1/4/5/9/10* quintuple mutant that lacks all subgroup I *SnRK2*s, and the *snrk2.1/4/5/7/8/9/10* septuple mutant that lacks all subgroups I and II *SnRK2*s. Similar to the *ABI1* overexpression lines, all *SnRK2* higher-order mutants showed reduced root length and smaller shoots compared with WT under 125 mM NaCl treatment (Fig. 5, A and B). Although mutants of different subgroups of *SnRK2*s showed similar growth defects, they exhibited distinct seed germination phenotypes under 175 mM NaCl. Germination of the ABA-insensitive *snrk2.2/3/6* triple mutant was less sensitive to NaCl, while the *snrk2.1/4/5/9/10* quintuple and *snrk2.1/4/5/7/8/9/10* septuple mutants were hypersensitive to NaCl during seed germination (Fig. 5, C and D, and fig. S6A). Expression of *SnRK2.1* and *SnRK2.4* driven by their native promoters complemented the defect of the *snrk2.1/4/5/7/8/9/10* mutant in seed germination under salt stress (Fig. 5, E and F, and fig. S6B). These results suggested that subgroup I SnRK2s differ from subgroup III SnRK2s and have pivotal roles in severe osmotic stress responses, especially during seed germination.

To further explore differences in transcriptional reprogramming during milder and severe osmotic stresses in relation to the roles of subgroup I and II SnRK2s, we performed transcriptomic analyses of WT and the *snrk2.1/4/5/7/8/9/10* mutant after treatments with 300 and 600 mM mannitol. Compared with WT, 71 genes (under control conditions), 65 genes (300 mM mannitol treatment), and 71 genes (600 mM mannitol) were down-regulated in the *snrk2.1/4/5/7/8/9/10* mutant (fig. S6, C and D). Among these genes, only 47 genes were specifically down-regulated in the *snrk2.1/4/5/7/8/9/10* mutant after 600 mM mannitol treatment (fig. S6D). Heatmaps show that most of the WT transcriptional responses to 600 mM mannitol were diminished in the *snrk2.1/4/5/7/8/9/10* mutant (Fig. 5G). We further analyzed gene expression changes in response to mild and severe osmotic stresses by quantitative reverse transcription polymerase chain reaction (RT-PCR). A group of stress-responsive genes, including *SWEET15* (*SAG29*) and *BGLU24*, were strongly induced in WT under 600 mM mannitol treatment, but this induction was reduced in the *snrk2.1/4/5/9/10* quintuple and *snrk2.1/4/5/7/8/9/10* septuple mutants or the ABA-insensitive *snrk2.2/3/6* mutant (Fig. 5H). In contrast, induction of the stress-responsive gene *DIN2* was markedly reduced in the *snrk2.1/4/5/9/10* quintuple mutant but weakly reduced in the *snrk2.2/3/6* triple mutant, while induction of the stress-responsive gene *LEA4*-*5* was markedly reduced in the *snrk2.2/3/6* triple mutant but not in the *snrk2.1/4/5/9/10* quintuple mutant (fig. S6E). Consistent with the repression of SnRK2s by clade A PP2Cs, induction of most of these stress-responsive genes was markedly reduced in the *ABI1* overexpression lines and elevated in the *abi1hab1pp2ca* triple mutant (Fig. 5I and fig. S6E). These results demonstrated that different subgroups of SnRK2s function together in regulating these stress-responsive genes under severe osmotic stress.

## DISCUSSION

**Fig. 6.**
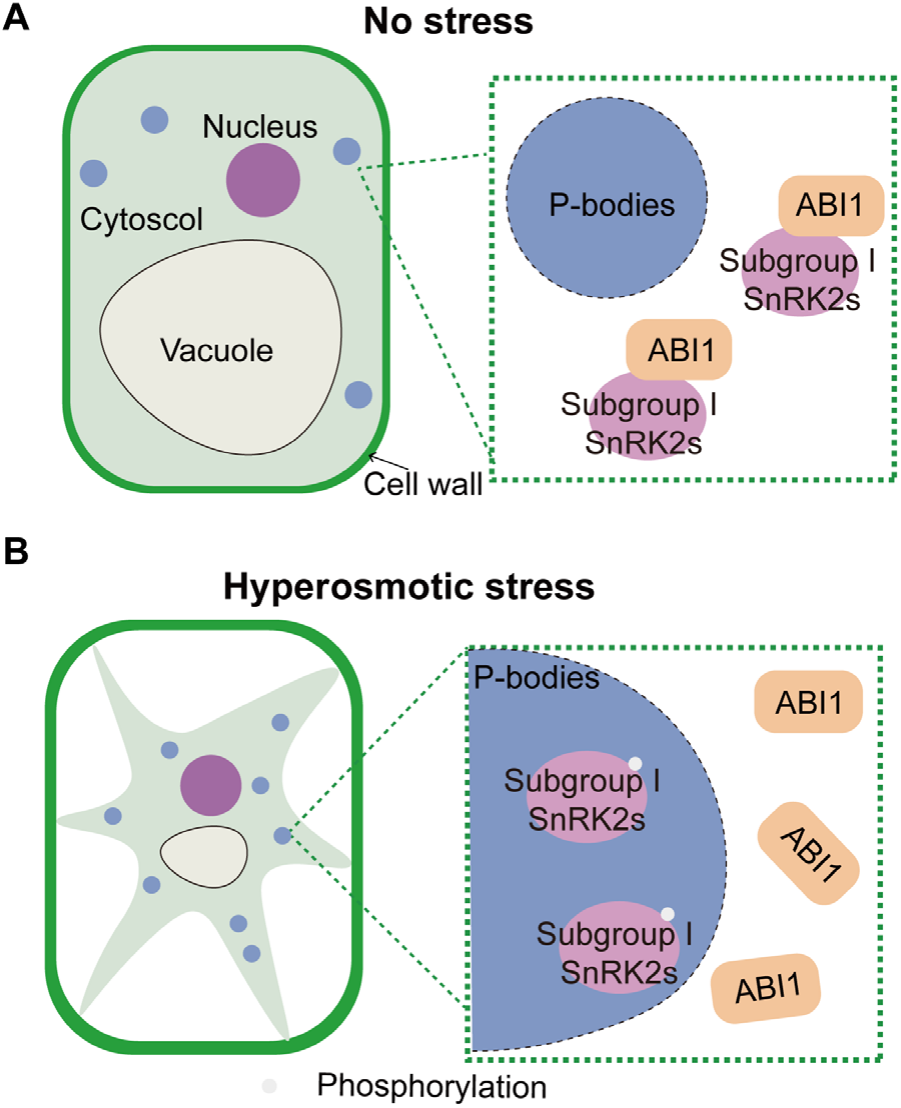
Working model for the spatial segregation–mediated release of subgroup I SnRK2s from ABI1 inhibition under severe osmotic stress. (**A**) Under unstressed conditions, the subgroup I SnRK2s are diffused in the cytosol and repressed by the negative regulator ABI1. (**B**) Upon severe osmotic stress, the subgroup I SnRK2s sense molecular crowding caused by the cell volume reduction, which prompts the formation of condensates via their C-terminal IDR domains. Formation of SnRK2 condensates leads to spatial segregation away from ABI1, leading to the release of SnRK2 from inhibition.

SnRK2s are core protein kinases in signaling downstream of osmotic stress and are repressed under unstressed conditions (*4*, *5*). SnRK2s are quickly released from inhibition upon sensing osmotic stress and subsequently activated in an ABA- and PYL-independent manner (*23*). However, how plants sense osmotic stress and how SnRK2s are released from inhibition remain unclear. Here, we report that SnRK2s are differentially released depending on the severity of the osmotic stress and identify the critical roles of the subgroup I SnRK2s in sensing cytosolic molecular crowding in plants under severe osmotic stress. Clade A PP2Cs are negative regulators of both subgroup I and III SnRK2s (Fig. 4 and fig. S4). Cell volume reduction upon hyperosmotic shock induces molecular crowding, which triggers phase separation of multivalent proteins (*26*). The subgroup I SnRK2s sense molecular crowding to form condensates via their C-terminal IDR domains (Figs. 2 and 3 and figs. S2 and S3), which in turn releases SnRK2s from PP2Cs by spatial segregation and initiates osmotic signaling in the condensates (Fig. 4 and figs. S4 and S5). In addition, condensation of the subgroup I SnRK2s does not require their kinase activities, supporting the notion that condensation precedes activation (Fig. 2, J and K). In contrast, few of the subgroup I SnRK2 molecules are released under milder osmotic stress (below or equal to isoosmotic solution), which may not greatly affect cell volume (Fig. 1 and fig. S1). Consistent with our hypothesis, hyperosmotic stress activates subgroup I SnRK2s but does not induce ABA accumulation in protoplasts that lack cell walls and turgor (*9*, *31*). The activated subgroup I SnRK2s phosphorylate the mRNA decapping activator VCS and the RNA-binding protein GRP8 (*6*, *24*), suggesting that those SnRK2s are closely associated with RNA processing. Subgroup I SnRK2s evolved in seed plants but are not found in lycophytes or mosses (*6*) and may contribute to seed or pollen physiology (*32*). Notably, seed germination of a higher-order mutant of the subgroup I *SnRK2*s is hypersensitive to NaCl, which contrasts the insensitive phenotype of the subgroup III *SnRK2* mutant (Fig. 5, C to F), suggesting unique roles of subgroup I SnRK2s in seed germination under stress. Moreover, the *snrk2.1/4/5/9/10* quintuple mutant exhibits normal growth under unstressed conditions (*4*). Our discovery therefore demonstrated that subgroup I SnRK2s function as sensors of molecular crowding during severe osmotic stress in plants (Fig. 6).

Unlike the subgroup I SnRK2s, the subgroup III SnRK2s are highly conserved from algae to seed plants (*5*) and are activated by milder osmotic stress that triggers turgor reduction. Subgroup III SnRK2s play critical roles in seed dormancy, plant growth, transcriptional responses, and stomatal regulation (*33*–*35*). Turgor reduction alters cell wall and membrane tension, which may be sensed and transduced via cell surface signaling (*1*, *36*–*38*). Although the sensing of turgor reduction is still unclear, recent findings indicate the critical role of BIK1 in releasing PP2C-mediated inhibition of SnRK2.6 under osmotic stress (*19*). Upon quick activation by osmotic stress, BIK1 phosphorylates SnRK2.6 at two conserved tyrosine residues, and this phosphorylation affects the docking of the tryptophan “lock” of PP2Cs into SnRK2.6 (*19*). Although the ancient member OST1 (open stomata 1) (also named SnRK2.6) has a conserved role in transcriptional regulation (*5*), whether PBL homologs in early land plants function in SnRK2 release is still an open question. During ABA signaling, the ABA-bound PYLs mimic and compete with SnRK2s for interaction with PP2Cs, therefore releasing SnRK2s from PP2C-mediated inhibition (*15*–*18*). The ABA-sensing tryptophan lock at the interface of PP2C-SnRK2 complexes plays a critical role during the SnRK2.6 release by PYLs or BIK1-mediated phosphorylation (*15*, *19*, *39*–*41*). SnRK2.6, but not SnRK2.2 and SnRK2.3, also forms condensates under severe osmotic stress (fig. S2K), suggesting that SnRK2.6 may also respond to macromolecular crowding under severe osmotic stress. The differential subcellular localization and activation of the SnRK2s in subgroups I and III may explain their functional differences in osmoregulation and support the hypothesis that osmotic stress responses are a cocktail of signaling mediated by multiple osmosensors (*1*).

Many macromolecules form condensates in response to molecular crowding under severe osmotic stress (*2*, *3*, *26*, *42*), which leads to self-assembly or segregation, triggering signal activation or attenuation. The dissociation of subgroup I SnRK2s from PP2Cs by condensation provides a plausible mechanism through which the core protein kinases sense molecular crowding and initiate signaling by spatial segregation in plants (Fig. 6). Although the IDR domains can form condensates in vitro (Fig. 3B), the other regions of the IDR-containing SnRK2 proteins are also required for condensation in vivo (fig. S3C), probably by mediating partner interactions or proper posttranslational modifications (*43*). The SnRK2 IDR domains have strong interactions with ABI1 (fig. S5). Although the IDR-deleted SnRK2.1 and SnRK2.4 lose interactions with ABI1, they are not activated in vivo (Fig. 3 and fig. S5). These SnRK2s may be subsequently activated by B4 RAFs that also localize to P-bodies under severe osmotic stress (*12*, *13*). Similarly, in human cells, condensation of signaling molecules promotes T cell receptor signaling by enriching protein kinases and excluding protein phosphatase during cell surface signaling (*44*). In contrast, the mammalian apoptosis signal–regulating kinase 3 (ASK3) and protein phosphatase 6 (PP6) condensate under hyperosmotic stress, which promotes PP6-mediated inactivation of ASK3 to control cell volume homeostasis (*45*). Therefore, condensation-mediated kinase disinhibition, activation, or inhibition may be a universal strategy among organisms. Future studies on the condensation of plant B4 RAFs and animal with-no-lysine kinases may further resolve their activation machinery upon severe osmotic stress (*3*, *12*, *13*).

Our study showed that plants perceive osmotic stress through cell surface signaling and direct activation of cytosolic signaling. Molecular crowding within the cytosol is a physiological signal as the cell volume reduces during severe osmotic stress. A subgroup of the core protein kinases in the SnRK2 family senses molecular crowding under severe osmotic stress, and rapid condensation of the IDR-containing kinases releases their inhibition by ABI1. This report elucidates the critical roles of phase separation in the spatial segregation of signaling molecules and signal initiation under severe osmotic stress.

## MATERIALS AND METHODS

### Plant materials

All plants are in the Col-0 ecotype background, unless indicated. The ABA signaling mutants *snrk2.2/3/6* and *abi1hab1pp2ca* have been described previously (*46*), and high-order *snrk2* mutants, including *snrk2.1/4/5/9/10* quintuple and *snrk2.1/4/5/7/8/9/10* septuple, have been generated previously (*4*). The transgene lines, including *35S:SnRK2.1/2/3/4/5/7/8/9/10-YFP*, *35S:ABI1/HAB1/PP2CA-YFP*, *35S:SnRK2.1^K33N^-YFP*, *35S:SnRK2.4^K33N^-YFP*, *35S:SnRK2.1IDR-YFP*, *35S:SnRK2.4IDR-YFP*, *35S:SnRK2.1*Δ*IDR-YFP*, *35S:SnRK2.4*Δ*IDR-YFP*, *35S:SnRK2.1-GFP*, *35S:SnRK2.4-GFP*, coexpressing *35S:SnRK2.1-YFP* and *35S:ABI1-CFP*, and coexpressing *35S:SnRK2.1-GFP* and *35S:mRFP-ABI1*, are in the Col-0 ecotype background. We also generated *35S:SnRK2.1-YFP* and *35S:SnRK2.4-YFP* lines in the *abi1hab1pp2ca* triple mutant background. The *SnRK2.1pro:SnRK2.1-GFP* and *SnRK2.4pro:SnRK2.4-GFP* transgenic seedlings were generated in the *snrk2.1/4/5/7/8/9/10* septuple mutant background. In addition, *ost1*-*3/SnRK2.6pro:SnRK2.6-GFP* and *Super:OST1-Myc* plants were used (*47*, *48*). All experiments were performed with transgenic lines in T3 generation.

### Plant growth conditions and treatments

Plants were grown in soil in a growth room at 22°C with 65 to 80% relative humidity under long-day conditions or on medium containing ½ MS nutrients (PhytoTech, M542) and 1% sucrose, pH 5.7, with 1.2% (w/v) agar (for vertical growth), in a growth chamber at 23°C with a 16-hour light/8-hour dark photoperiod and 40% relative humidity. *N. benthamiana* was grown in soil in a growth room under a 16-hour light/8-hour dark photoperiod at 23°C. Seeds were surface sterilized, grown vertically on ½ MS medium, and kept at 4 to 8°C for 2 days.

For SnRK2 activity analysis, seedlings were grown vertically for 7 days and then transferred to a liquid ½ MS medium for pretreatment. After 2 hours, the seedlings were transferred to the liquid ½ MS medium containing 50 μM ABA or different concentrations of mannitol (0.3, 0.6, and 0.8 M). After being treated for indicated times, the samples were briefly blotted up and quickly frozen with liquid nitrogen for further analysis. For transcriptional analysis, seedlings were grown vertically for 9 days and then treated with different concentrations of mannitol (0.3 and 0.6 M) or control (½ MS) for 6 hours, and the samples were briefly blotted up and quickly frozen with liquid nitrogen for further analysis.

### Plasmid construction and plant transformation

The entire coding sequences of *SnRK2s* and *PP2Cs*, including *SnRK2.1*, *SnRK2.2*, *SnRK2.3*, *SnRK2.4*, *SnRK2.5*, *SnRK2.7*, *SnRK2.8*, *SnRK2.9*, *SnRK2.10*, *ABI1*, *HAB1*, and *PP2CA*, were amplified and cloned into *pCAMBIA1300* vectors to generate the *35S:SnRK2s-YFP* and *35S:PP2Cs-YFP* constructs, respectively. The entire coding sequence of *ABI1* was amplified and cloned into *pCAMBIA1300* and *pCAMBIA3301* to generate *35S:ABI1-CFP* and *35S:mRFP-ABI1*, respectively. The resultant plasmids were confirmed by sequencing and transformed into the Col-0 background. To generate *SnRK2pro:SnRK2-GFP* transgenic plants, the genomic sequences of *SnRK2.1* [a 1236–base pair (bp) promoter and 2330 bp of the entire coding region without the stop codon] and *SnRK2.4* (a 1975-bp promoter and 2118 bp of the entire coding region without the stop codon) were cloned into the *pCAMBIA1305* vector. The resultant plasmids were confirmed by sequencing and transformed into the *snrk2.1/4/5/7/8/9/10* septuple mutant background.

All transgenic plants were generated using the *Agrobacterium*-mediated flower-dip transformation method and were screened for hygromycin resistance, which was then confirmed by Western blot using an anti-GFP antibody. Transgenic lines coexpressing combinations of *SnRK2.1-YFP* and *ABI1-CFP* or *SnRK2.1-GFP* and *mRFP-ABI1* were screened for hygromycin and Basta resistance.

### Generation of phosphosite-specific antibody and immunoblotting

Phosphosite-specific rabbit antibodies were generated by ABclonal Technology. The high-purity modified peptide SILHSRPK(S-p)TVGT-C, purified by high-performance liquid chromatography, was used as an antigen to generate polyclonal anti-phosphosite antibodies for Ser^158^ in SnRK2.1 (anti-phospho-S158-SnRK2.1 antibody). The phosphorylation site–specific antibody was purified twice using the nonmodified peptide SILHSRPKSTVGT-C (which absorbed the nonspecific antibodies) and modified peptide SVLHSQPK(S-p)TVGT-C (which absorbed antibodies recognizing the phosphorylation of Ser^175^ in SnRK2.6). The other antibodies used in the project are the polyclonal anti-phosphorylation site antibodies for Ser^175^ (ABclonal, AP1481) or Ser^171^ in SnRK2.6 described previously (*10*, *23*).

Total protein extraction and Western blots were performed as previously described (*23*), with modifications. Generally, the proteins were extracted using an extraction buffer [40 mM tris-HCl, pH 7.5, 200 mM NaCl, 2 mM EDTA, 2 mM EGTA, 2 mM Na_3_VO_4_, 2 mM NaF, 20 mM β-glycerophosphate, leupeptin (4 mg/ml), antipain (4 mg/ml), aprotinin (4 mg/ml), 0.2% Tween 20, and 1× InStab Protease Cocktail]. For Western blots, the membranes were incubated at 4°C overnight in 5% bovine serum albumin (BSA) containing 1:2000 diluted phosphosite-specific antibody for Ser^158^ of SnRK2.1. After being washed three times (10 min each) with tris-buffered saline with Tween 20 (TBST), the membranes were incubated for 2 hours at room temperature in TBST with 3% BSA containing 1:10,000 diluted goat antirabbit horseradish peroxidase–conjugated (HRP) antibodies (Bio-Rad, 172-1019) and then washed and detected using Lumi-Light Western Blotting Substrate (shared, 12015196001) with 30 to 60 s of exposure.

### Microscopy

For imaging of SnRK2s and DCP1 in tobacco leaves, a small disc from a tobacco leaf was soaked in water with or without 500 mM mannitol for 60 min. For imaging of SnRK2s in *Arabidopsis*, a 4-day-old vertically grown seedling was soaked in water with or without 200 mM NaCl for 20 min, different concentrations of mannitol (300, 400, 500, 600, and 800 mM) for 10 min, or 50 μM ABA for 30 min. The seedling was then transferred onto a slide and imaged on a Zeiss LSM880 confocal laser microscope. Imaging of *Arabidopsis* was performed using a ×40/1.20–numerical aperture (NA) water objective. CFP (cyan fluorescent protein) was excited at 456 nm and detected at 475 to 525 nm, GFP was excited at 488 nm and detected at 500 to 550 nm, YFP was excited at 514 nm and detected at 530 to 580 nm, and mCherry and mRFP were excited at 561 nm and detected at 605 to 645 nm. The foci numbers are calculated with FIJI/ImageJ.

### Fluorescence recovery after photobleaching and time-lapse imaging

For the in vivo experiments, the seedling was transferred onto a slide and imaged on a Leica TCS SP8 laser scanning confocal microscope using a ×63/1.20-NA water objective. A SnRK2.1-GFP condensate region was bleached using a 488-nm laser pulse. Recovery was recorded every 5 s for a total of 60 s after bleaching. For time-lapse imaging, seedlings were treated with 500 mM sorbitol and immobilized on slides during imaging. GFP was excited at 488 nm and detected at 500 to 550 nm. Images were acquired with a ×63/1.20-NA water objective. Analysis of the recovery curves and time lapse was performed with FIJI/ImageJ.

### In vitro phase separation assay

The in vitro phase separation assay was performed as described (*49*). The *pMal-p2xM* and *pET32a* vectors were used for the expression of recombinant proteins. The maltose-binding protein (MBP) tags of fusion proteins, including MBP-His-SnRK2.1-GFP, MBP-His-SnRK2.4-GFP, MBP-His-SnRK2.1ΔIDR-GFP, and MBP-His-SnRK2.4ΔIDR-GFP, were cleaved with TEV-His protease overnight in 4°C. After the proteolytic cleavage, MBP-His and TEV-His were removed by Ni-NTA beads. The TEV (tobacco etch virus)–mediated cleavage was evaluated using SDS–polyacrylamide gel electrophoresis (SDS-PAGE) analysis. SnRK2.1IDR-GFP and SnRK2.4IDR-GFP were directly purified by Ni-NTA beads after being expressed using the *pET32a* vector. Various concentrations of PEG6000 were then added to the protein samples. Droplets were visualized using a Zeiss LSM880 confocal microscope with a ×100/1.40-NA oil objective.

### In vitro dephosphorylation assay

The coding sequences of *SnRK2.1* and *SnRK2.4* were amplified and cloned into the *pMal-p2xM* vector to obtain *His-MBP-SnRK2.1-GFP* and *His-MBP-SnRK2.4-GFP* constructs, respectively. The coding sequence of *ABI1* was amplified and cloned into the *pGEX-6P-1* vector to obtain the *GST-ABI1* construct. The resultant plasmids were confirmed by sequencing and used to express recombinant proteins in *Escherichia coli*.

The in vitro kinases assay was performed as described (*23*) in the presence of protein phosphatase. *GST-ABI1*, *His-MBP-SnRK2.1-GFP*, and *His-MBP-SnRK2.4-GFP* were incubated in 25 μl of reaction buffer [50 mM tris-HCl, pH 7.5, 20 mM MgCl_2_, 0.25 mM dithiothreitol, 1 μM adenosine 5′-triphosphate (ATP), and 5 mCi (γ-^32^P) ATP] at 30°C. After incubation for 2 hours, the proteins were separated by SDS-PAGE. After electrophoresis, the gel was dried under vacuum at 80°C for 1 hour on filter paper and then exposed to a phosphor-imager overnight. Radioactivity was detected with a Personal Molecular Imager (Bio-Rad, Hercules, CA) or Typhoon biomolecular imager (GE).

### Immunoprecipitated kinase assay

The immunoprecipitated kinase assays of SnRK2s and SnRK2ΔIDRs were performed as previously described with some modifications (*23*). Nine-day-old seedlings were treated with or without 0.6 mannitol for the indicated times. Samples (about 0.5 g) were collected and ground in liquid nitrogen. The total protein was extracted in 1.5 ml of 2× immunoprecipitation (IP) buffer. After being centrifuged at 14,500*g* for 30 min, the supernatants were incubated with GFP beads for 3 hours at 4°C. After incubation, the beads were washed three times with 1× IP buffer, followed by kinase buffer (25 mM tris-HCl, pH 7.5, 10 mM MgCl_2_, and 0.25 mM dithiothreitol) one more time. Then, the GFP beads were incubated in kinase buffer with the ATP analog ATPγS at room temperature for 30 min for in vitro phosphorylation assays, followed by the *p*-nitrobenzyl mesylate (PNBM) alkylation. The proteins were separated by SDS-PAGE. After electrophoresis, the gel was transferred to a polyvinylidene difluoride (PVDF) membrane for Western blots. The anti-thiophosphate ester antibody (Abcam, ab92570, 1:10,000) was used to detect the phosphorylated proteins. The anti-GFP antibody was used as a loading control.

### Yeast two-hybrid (Y2H) assay

The coding sequences of *GUS*, *SnRK2.1*, *SnRK2.4*, *SnRK2.5*, *SnRK2.9* and *SnRK2.10*, and truncated SnRK2.1/2.4 were amplified by PCR and cloned into the *pGBKT7* vector, while the coding sequences of *ABI1* was amplified by PCR and cloned into the *pGADT7* vector. All constructs were transformed into the Y2H Gold strain using the LiAc/PEG method. Yeast colonies were selected on the SD medium lacking Leu and Trp (-L-W) and transferred to the SD medium lacking Leu, Trp, and His (-L-W-H). Dilutions of saturated yeast cultures were spotted onto the selection medium. Photographs were taken after 3 days of incubation at 28°C.

### BiFC assay

The full-length coding sequences of *ABI1*, *At4g33920*, and full-length and truncated *SnRK2s* were cloned into *pCAMBIA1300S-YC* and *pCAMBIA1300S-YN* to generate the *SnRK2s-nYFP*, *ABI1-cYFP*, and *At4g33920-cYFP* constructs, which were agro-infiltrated into the leaves of *N. benthamiana*. The plants were grown in the dark for 48 hours after infiltration, and BiFC fluorescence signals were observed using a ZEISS LSM880 confocal laser-scanning microscope.

### Split-LUC complementation assay

The coding sequences of *GUS*, *SnRK2.1*, and *SnRK2.4* were amplified by PCR and cloned into the *pCAMBIA-35S-nLUC* vector, while the coding sequences of *ABI1*, *HAB1* and *PP2CA* were amplified by PCR and cloned into the *pCAMBIA-35S-cLUC* vector. The split-LUC complementation assay was performed by transient expression of indicated combinations of constructs in tobacco leaves through *Agrobacterium*-mediated infiltration. Two days after infiltration, LUC activity was detected with a charge-coupled device camera by applying firefly d-luciferin (NanoLight). *GUS*-*nLUC* was used as a negative control.

### Measurement of water loss

The detached rosettes of 4-week-old plants were placed in plastic weighing dishes and left on the laboratory bench with light. Fresh weight was weighed at the indicated time points. Water loss was expressed as a percentage of initial fresh weight.

### Measurement of osmolarity

Seedlings are collected and shattered in liquid nitrogen. After centrifuging at 12,000 rpm for 30 min, the supernatants are used to measure osmolarity with an osmometer (Wescor 5600R).

### Seed germination and plant growth under osmotic stress

Seeds were surface sterilized for 10 min in 5% sodium hypochlorite and then rinsed three times in double-distilled water. For seed germination assays, sterilized seeds were grown horizontally on ½ MS with or without 175 mM mannitol and kept at 4°C for 3 days. Radicle emergence was analyzed 0 to 7 days after placing the plates at 23°C. Photographs of seedlings were taken on day 7. For plant growth assays, sterilized seeds were grown vertically on a ½ MS medium and kept at 4°C for 48 hours. Seedlings were grown vertically for 4 to 5 days and then transferred to media with or without 100 mM NaCl, 125 mM NaCl, or 10 μM ABA. Photographs of seedlings were taken on the 12th day after seedlings were transferred, and root length was measured.

### RNA extraction and quantitative RT-PCR

Total RNA was extracted from 9-day-old seedlings using TRIzol (Invitrogen). Reverse transcription reactions were performed with 1 μg of total RNA using HifairIII First Strand cDNA Synthesis SuperMix (Yeasen, Shanghai, China, 11141ES60). Quantitative RT-PCR was then performed for 40 cycles. The real-time qPCR (quantitative PCR) assay was performed with 2× Universal SYBR Green Fast qPCR Mix (Abclonal, China, RK21203) according to the manufacturer’s instructions. The primers used for real-time PCR are listed in data S1.

### mRNA sequencing and data analysis

Three independent RNA samples were used as biological replicates in each RNA sequencing experiment. RNA extraction, RNA purification, reverse transcription, library construction, and sequencing were performed at Shanghai Majorbio Bio-pharm Biotechnology Co., Ltd. (Shanghai, China) according to the manufacturer’s instructions (Illumina, San Diego, CA). To identify differential expression genes between Col-0 and the *snrk2.1/4/5/7/8/9/10* septuple mutant, the expression level of each gene was calculated according to the transcripts per million reads method. Differential expression analysis was performed using DESeq2. Functional-enrichment analyses, including GO (Gene Ontology) and KEGG (Kyoto Encyclopedia of Genes and Genomes), were performed to identify differential expression genes that were notably enriched in GO terms and metabolic pathways at *P* < 0.05 compared with the whole-transcriptome background. The RNA sequencing data are shown in data S2.

### Phylogenetic tree and structure prediction of SnRK2s

The evolution of SnRK2s was analyzed using the maximum likelihood method in MEGA-X. The IUPred3 algorithm made predictions of the disordered regions.

### Quantification and statistical analysis

Statistical parameters are reported in the figures and figure legends. Statistical analysis was performed using GraphPad Prism 8. The two-tailed Student’s *t* test was used to analyze the statistical significance between the two groups, and asterisks indicate the statistical significance: *P < 0.05; **P < 0.01; ***P < 0.001; ****P < 0.0001. One-way analysis of variance (ANOVA) and two-way ANOVA followed by a Tukey test were used to analyze the statistical significance for more than two groups. Colocalization analysis was performed with FIJI/ImageJ.
