# Supplementary material for: SnRK2 kinases sense molecular crowding and form condensates to disrupt ABI1 inhibition
Source: Sci Adv. 2025 Jan 29;11(5):eadr8250. doi: 10.1126/sciadv.adr8250 (PMC11777248; doi:10.1126/sciadv.adr8250)
Supplement: Supplementary file 1 — Figs. S1 to S6 Legend for movie S1 Legends for data S1 and S2 [file sciadv.adr8250_sm.pdf]

Science Advances
AAAS

Supplementary Materials for

SnRK2 kinases sense molecular crowding and form condensates to disrupt ABI1 inhibition

Xian-Ping Yuan and Yang Zhao

Corresponding author: Yang Zhao, yangzhao@psc.ac.cn

Sci. Adv. **11**, eadr8250 (2025)
DOI: 10.1126/sciadv.adr8250

The PDF file includes:

Figs. S1 to S6
Legend for movie S1
Legends for data S1 and S2

Other Supplementary Material for this manuscript includes the following:

Movie S1
Data S1 and S2

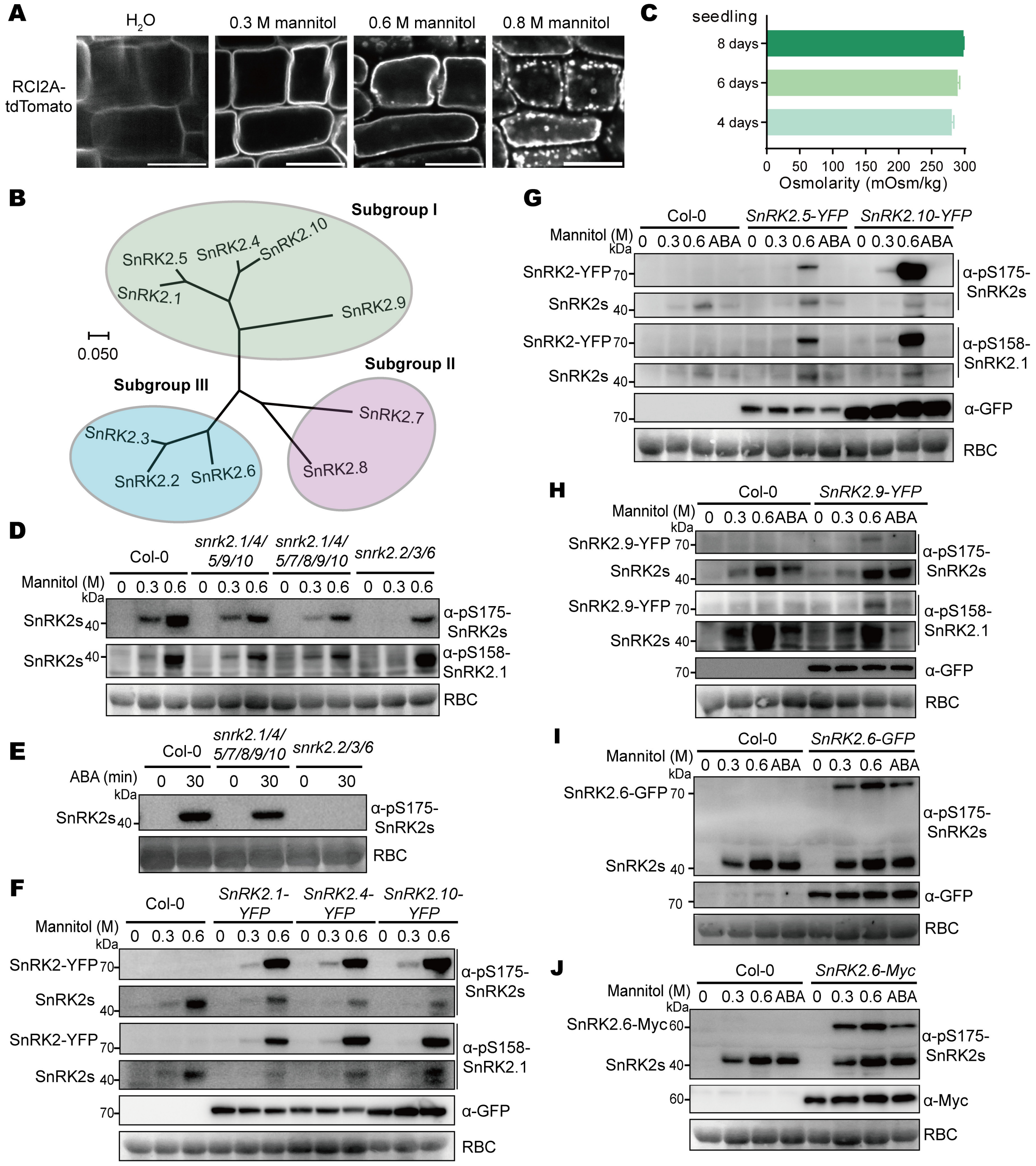

Fig. S1. Activation of SnRK2s in subgroups III and I under different levels of osmotic stress and ABA.

(**A**) The cellular responses of root columella cells to 10-minute treatments with 0.3 M, 0.6 M, or 0.8 M mannitol were monitored using the plasma membrane marker RCI2A-tdTomato. The amplified image is shown in Fig. 1A. Scale bar, 10 μm. (**B**) Phylogenetic tree of SnRK2s. (**C**)

The osmolarity of wild-type *Arabidopsis* seedlings grown on ½ MS medium for different days after germination. (**D**) Phosphorylation of SnRK2s in 7-day-old WT or *snrk2* mutants, namely *snrk2.2/3/6*, *snrk2.1/4/5/9/10* and *snrk2.1/4/5/7/8/9/10*, after treatment with 0.3 M or 0.6 M mannitol for 30 min. (**E**) Phosphorylation of SnRK2s in 7-day-old WT and *snrk2.2/3/6* and *snrk2.1/4/5/7/8/9/10* mutants after treatment with 50 μM ABA for 30 min. (**F**) Phosphorylation of YFP-tagged SnRK2.1, SnRK2.4 and SnRK2.10 (about 70 kDa) and endogenous SnRK2s (about 40 kDa) after treatment with 0.3 M or 0.6 M mannitol for 30 min, in 7-day-old WT or *SnRK2-YFP* overexpression lines. (**G** and **H**) Phosphorylation of YFP-tagged SnRK2.5 and SnRK2.10 (about 70 kDa) (G), YFP-tagged SnRK2.9 (about 70 kDa) (H), and endogenous SnRK2s (about 40 kDa), after 30 min treatment with mannitol (0.3 M or 0.6 M) or 50 μM ABA in 7-day-old WT or *SnRK2-YFP* overexpression lines. (**I** and **J**) Phosphorylation of SnRK2.6-GFP (I, about 70 kDa) and SnRK2.6-Myc (J, about 60 kDa) and endogenous SnRK2s (about 40 kDa), after 30 min treatments with mannitol or 50 μM ABA in 7-day-old seedlings of WT, *SnRK2.6pro:SnRK2.6-GFP* in the *snrk2.6* mutant background, or *SnRK2.6-Myc* overexpression in the *snrk2.6* mutant background. SnRK2 phosphorylation was detected with anti-phospho-S175-SnRK2s (D-J), and anti-phospho-S158-SnRK2.1 antibodies (D, F-H). Protein loading was detected by anti-GFP antibody (F-I), anti-Myc antibody (J), and Ponceau S staining for Rubisco (RBC) (D-J). All experiments were repeated at least three times with similar results.

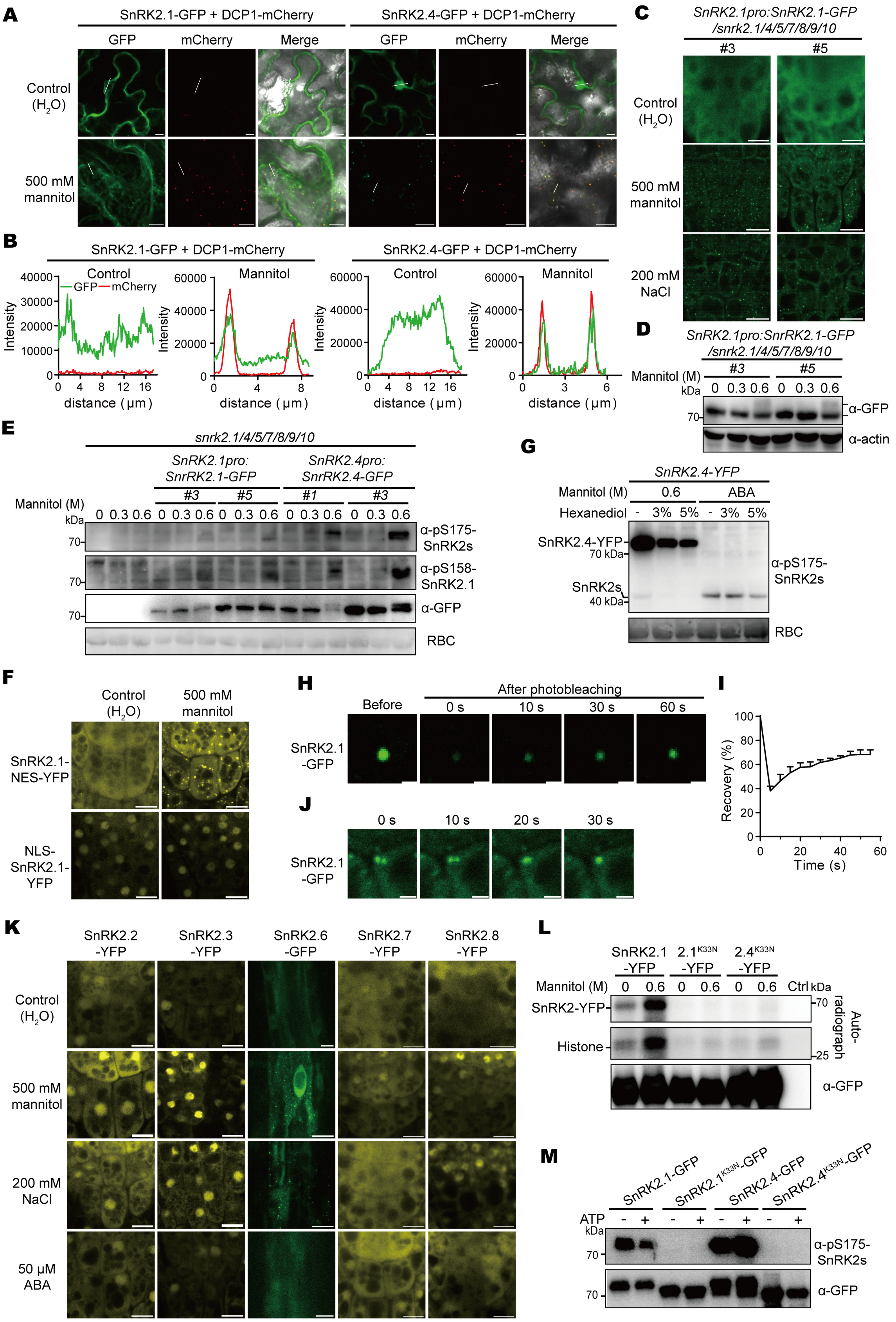
A
SnRK2.1-GFP + DCP1-mCherry
SnRK2.4-GFP + DCP1-mCherry
GFP
mCherry
Merge
Control (H_2O)
500 mM mannitol
B
Control
Mannitol
GFP
mCherry
Intensity
distance (μm)
C
SnRK2.1pro:SnRK2.1-GFP /snrk2.1/4/5/7/8/9/10
#3
#5
200 mM NaCl
D
Mannitol (M)
kDa
α-GFP
α-actin
E
snrk2.1/4/5/7/8/9/10
SnRK2.1pro: SnRK2.1-GFP
SnRK2.4pro: SnRK2.4-GFP
#1
α-pS175-SnRK2s
α-pS158-SnRK2.1
RBC
F
SnRK2.1-NES-YFP
NLS-SnRK2.1-YFP
G
SnRK2.4-YFP
ABA
Hexanediol
SnRK2s
40 kDa
70 kDa
H
Before
After photobleaching
0 s
10 s
30 s
60 s
SnRK2.1-GFP
I
Recovery (%)
Time (s)
J
20 s
K
SnRK2.2-YFP
SnRK2.3-YFP
SnRK2.6-GFP
SnRK2.7-YFP
SnRK2.8-YFP
50 μM ABA
L
SnRK2.1-YFP
2.1K33N-YFP
2.4K33N-YFP
Ctrl
SnRK2-YFP
Histone
Auto-radiograph
M
SnRK2.1-GFP
SnRK2.1K33N-GFP
SnRK2.4-GFP
SnRK2.4K33N-GFP
ATP

Fig. S2. Severe osmotic stress triggers condensation of SnRK2.1, 2.4, and 2.6, but not SnRK2.2 and 2.3.

(**A**) Subcellular localization of SnRK2.1/2.4-GFP and DCP1-mCherry in *N. benthamiana* leaves expressing both DCP1-mCherry and either SnRK2.1-GFP or SnRK2.4-GFP before and after treatment with 500 mM mannitol for 60 min. (**B**) Intensity scale values of co-localization on the white lines in (A). (**C**) Representative confocal microscopic images of GFP-tagged SnRK2.1 in root tip cells of *SnRK2.1pro:SnRK2.1-GFP* transgenic seedlings in the *snrk2.1/4/5/7/8/9/10* septuple mutant background, after treatments with 500 mM mannitol for 10 min or 200 mM NaCl for 20 min. Two lines are shown. (**D**) Band shift of SnRK2.1-GFP in 7-day-old *SnRK2.1pro:SnRK2.1-GFP* transgenic seedlings in the *snrk2.1/4/5/7/8/9/10* septuple mutant background, after treatments with 0.3 M or 0.6 M mannitol for 30 min. SnRK2.1-GFP was detected by an anti-GFP antibody (top). Actin was used as the loading control (bottom). Two lines are shown. (**E**) Phosphorylation of GFP-tagged SnRK2.1 and 2.4 (about 70 kDa) after 30 min treatments with 0.3 M and 0.6 M of mannitol in 7-day-old *SnRK2pro:SnRK2-GFP* transgenic seedlings in the *snrk2.1/4/5/7/8/9/10* septuple mutant background. SnRK2 phosphorylation was detected with anti-phospho-S175-SnRK2s and anti-phospho-S158-SnRK2.1 antibodies. Protein loading was detected by anti-GFP antibody and Ponceau S staining for Rubisco (RBC). Two lines are shown. (**F**) Representative confocal images of YFP-tagged SnRK2.1 fused with either a nuclear localization signal (NLS) or a nuclear export signal (NES) in root tip cells of transgenic *Arabidopsis* overexpressing *SnRK2-NES-YFP* or *NLS-SnRK2.1-YFP* driven by *35S* promoter after treatments with 500 mM mannitol for 10 min. (**G**) Phosphorylation of YFP-tagged SnRK2.4 (about 70 kDa) and endogenous SnRK2s (about 40 kDa) in 7-day-old *SnRK2.4-YFP* overexpression lines after treatment with 0.6 M mannitol or 50 μM ABA, with 0%, 3% or 5% 1,6-hexanediol co-treatments. SnRK2 phosphorylation was detected with anti-phospho-S175-SnRK2s antibody. Protein loading was detected by Ponceau S staining for RBC. (**H**) Fluorescence recovery after photobleaching (FRAP) of SnRK2.1-GFP condensates formed in *Arabidopsis* root tips of *SnRK2.1-GFP* overexpression lines driven by *35S* promoter. Time 0 s indicates the time of the photobleaching pulse. (**I**) Plot showing the time course of the recovery after photobleaching SnRK2.1-GFP condensates. Error bars indicate SEM (n = 3 independent foci). (**J**) Increasing size of SnRK2.1-GFP droplets over time in *Arabidopsis* root tips of *SnRK2.1-GFP* overexpression lines after addition of 500 mM mannitol. (**K**) Confocal microscopic images of YFP-tagged SnRK2.2, 2.3, 2.7 and 2.8 or GFP-tagged SnRK2.6 in *Arabidopsis* root tip cells of either *SnRK2.2/2.3/2.7/2.8-YFP* overexpression or *SnRK2.6pro:SnRK2.6-GFP* transgenic seedlings in the *snrk2.6* mutant background, after treatments with 500 mM mannitol for 10 min, 200 mM NaCl for 20 min, or 50 μM ABA for 30 min. YFP-tagged SnRK2.2, 2.3, 2.7 and 2.8 were visualized in the root columella cells, while SnRK2.6-GFP was visualized in the root pericycle. (**L**) Autophosphorylation and transphosphorylation activities of wild-type and "kinase-dead" SnRK2-YFP immunoprecipitated from the *SnRK2.1-YFP* and *SnRK2.4-YFP* overexpression lines after 0.6 M mannitol treatment for 30 min. Autoradiography (top) exhibits autophosphorylation and transphosphorylation of histone. Loading of SnRK2-YFP proteins was detected with anti-GFP antibody. **(M)** Phosphorylation of wild-type and "kinase-dead" forms of SnRK2.1 and SnRK2.4 recombinant proteins was detected with anti-phospho-S175-SnRK2s antibody. Loading of GFP-fused SnRK2.1/2.4 was detected with anti-GFP antibody. Scale bars in [(A), (C), (F), (K)] were 10 μm and scale bars in (H) and (J) were 1 μm.

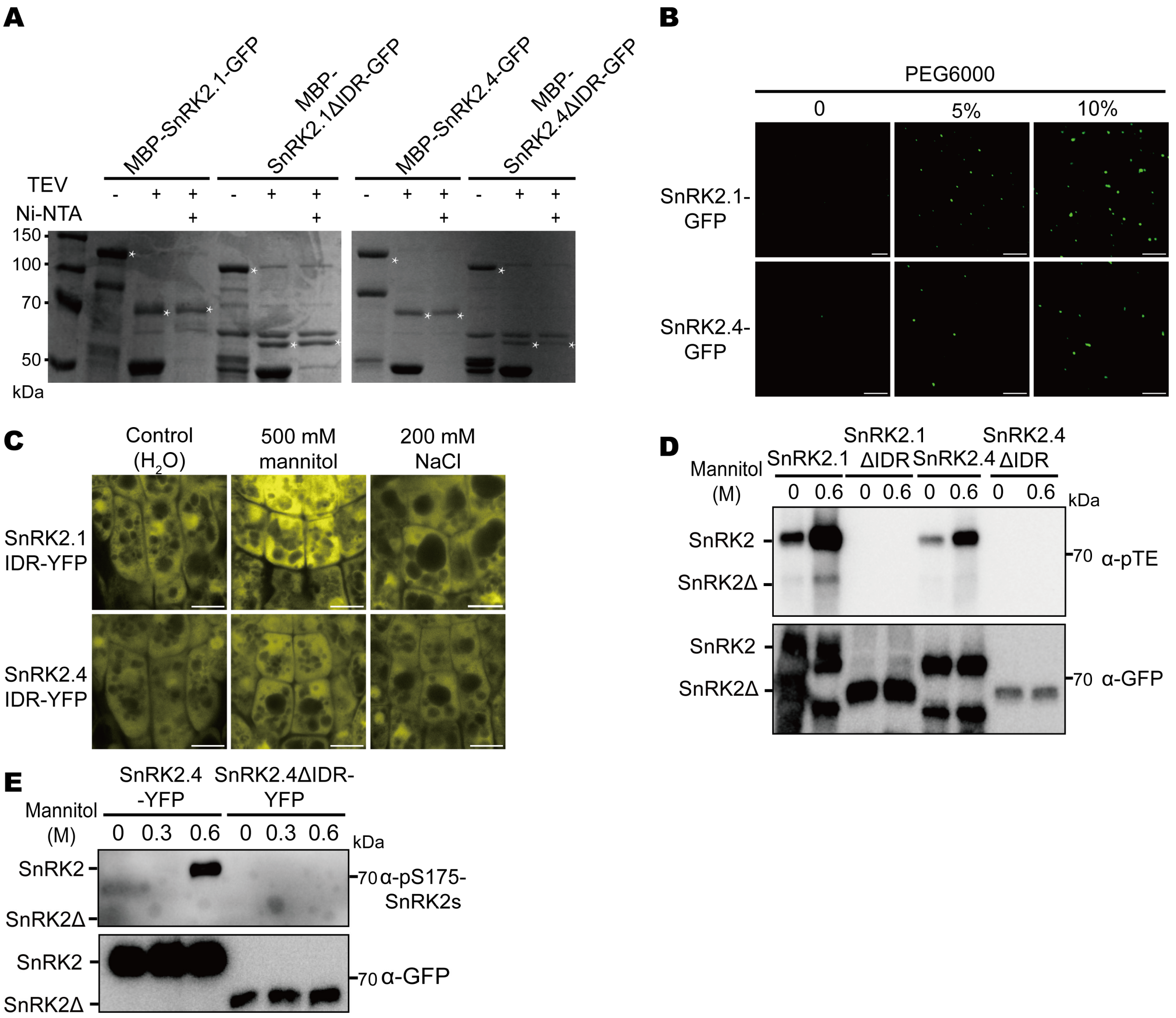

Fig. S3. The IDR of subgroup I SnRK2s is required for condensation and activation.

(**A**) Coomassie staining of indicated proteins before and after TEV cleavage to remove the MBP tag. Asterisks indicate the target proteins. (**B**) *In vitro* phase separation assay of SnRK2.1 and SnRK2.4 recombinant proteins in the presence of indicated concentrations of PEG6000. Scale bars, 5 µm. (**C**) Confocal microscopic images of *Arabidopsis* root tip cells expressing YFP-tagged SnRK2.1-IDR and SnRK2.4-IDR after 500 mM mannitol treatment for 10 min, or 200 mM NaCl for 20 min. Scale bars, 10 µm. (**D**) Autophosphorylation activity of YFP-tagged SnRK2.1 and SnRK2.4 (about 70 kDa) and their truncations (about 60 kDa) immunoprecipitated from the respective *SnRK2-YFP* overexpression lines after 0.6 M mannitol treatment for 30 min. After the PNBM alkylation reaction, the thiophosphate ester groups on the substrate were detected by the anti-thiophosphate ester antibody (α-pTE). Loading of the SnRK2-YFP proteins was detected by an anti-GFP antibody. (**E**) Phosphorylation of YFP-tagged SnRK2.4 (about 70 kDa) and its truncation (about 60 kDa), immunoprecipitated from the respective *SnRK2-YFP* overexpression lines after 30 min treatments with different concentrations of mannitol (0.3 and 0.6 M). SnRK2 phosphorylation was detected with anti-phospho-S175-SnRK2s antibody. Protein

loading was detected by an anti-GFP antibody. All experiments were repeated at least three times with similar results.

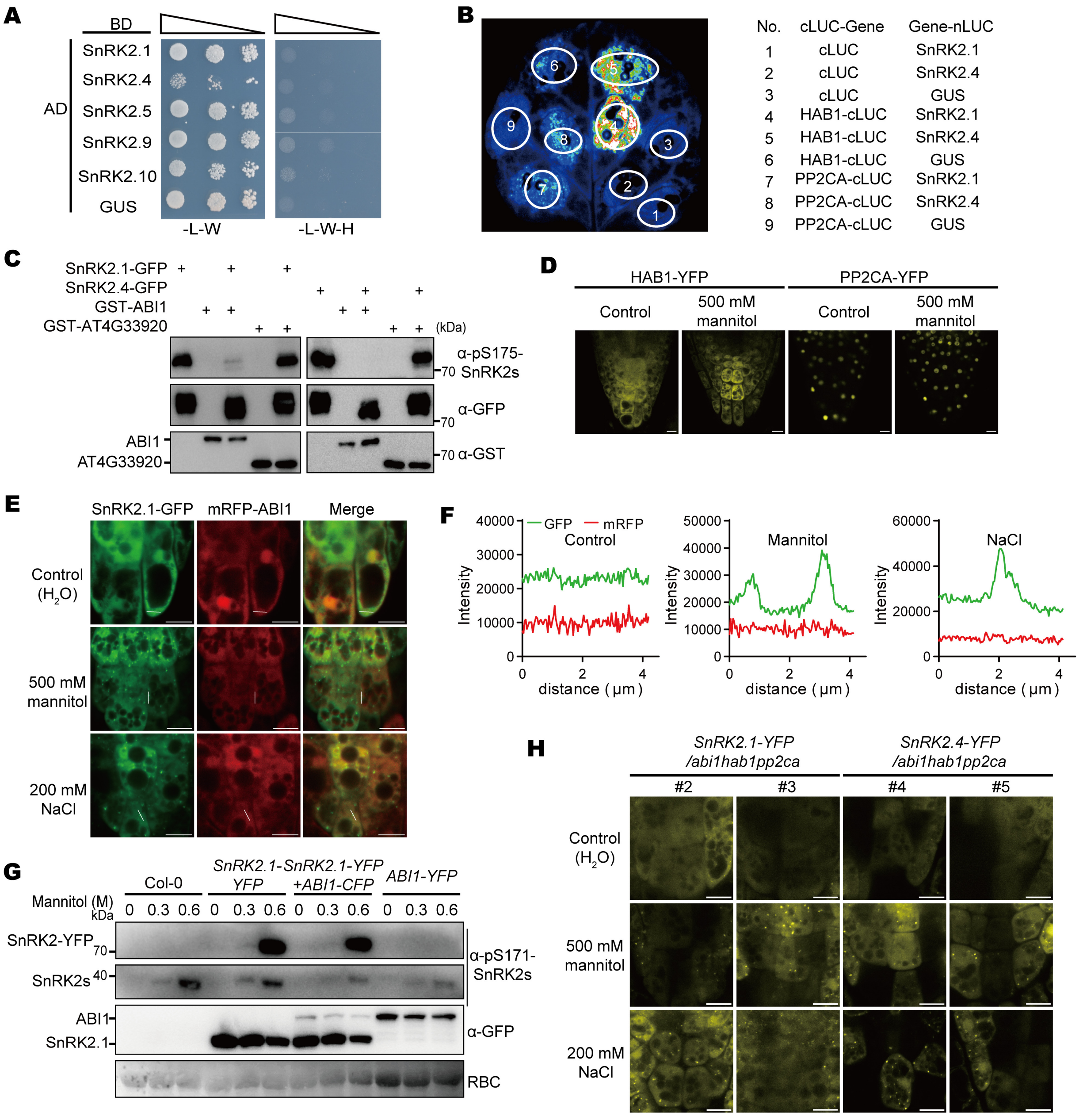

Fig. S4. Spatial segregation via condensation releases SnRK2 kinases from ABI1.

(**A**) Negative controls in Y2H assay. AD fusions with BD-SnRK2s were used as negative controls. (**B**) HAB1-SnRK2 and PP2CA-SnRK2 interactions in *N. benthamiana* leaves using split-LUC. GUS-nLUC was used as a negative control. (**C**) Dephosphorylation and inhibition of SnRK2.1 and SnRK2.4 by recombinant GST-ABI1. SnRK2 phosphorylation was detected with anti-phospho-S175-SnRK2s antibody. Protein loading was detected by anti-GFP and anti-GST antibodies. At4g33920 was used as a negative control. (**D**) Confocal images of *Arabidopsis* root tip cells overexpressing HAB1/PP2CA-YFP after treatment with 500 mM mannitol for 10 min. Scale bars, 10 µm. (**E**) Representative confocal microscopic images of *Arabidopsis* root tip cells co-expressing SnRK2.1-GFP and mRFP-ABI1 after treatment with either 500 mM mannitol for

10 min or 200 mM NaCl for 20 min. Scale bars, 10 µm. (**F**) Intensity scale values of co-localization on the white lines in (E). (**G**) Phosphorylation of YFP-tagged SnRK2.1 (about 70 kDa) and endogenous SnRK2s (about 40 kDa) after 30 min treatments with different concentrations of mannitol (0.3 and 0.6 M) in 7-day-old transgenic lines either overexpressing *SnRK2.1-YFP* or *ABI1-YFP* or co-expressing *SnRK2.1-YFP* and *ABI1-CFP*. (**H**) Representative confocal images of YFP-tagged SnRK2.1/2.4 in *Arabidopsis* root tip cells of *SnRK2-YFP* overexpression lines in the background of the *abi1hab1pp2ca* higher order mutant after treatments with either 500 mM mannitol for 10 min or 200 mM NaCl for 20 min. Scale bars, 10 µm.

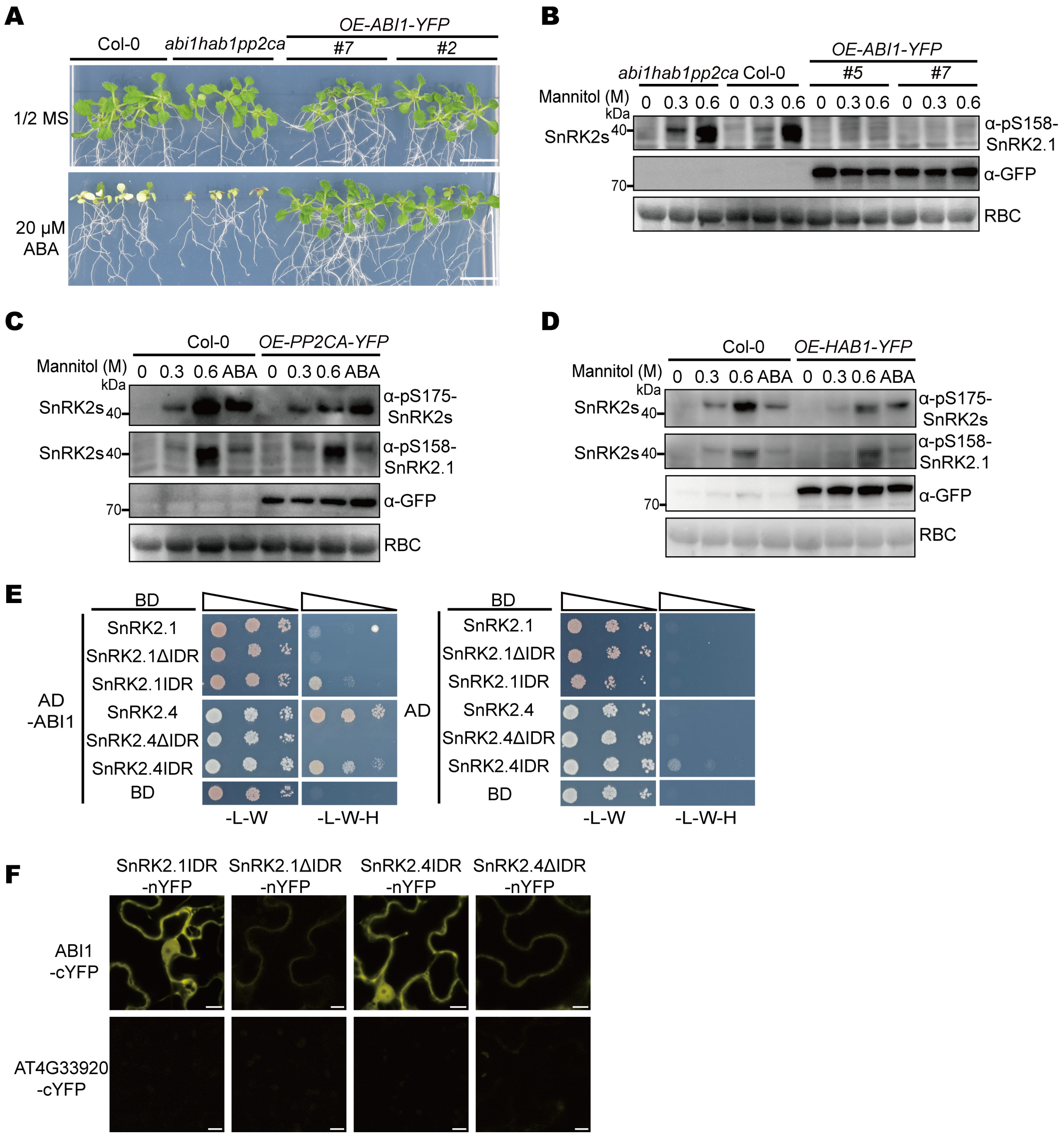

Fig. S5. PP2Cs are negative regulators of SnRK2s.

(**A**) WT (Col-0), *abi1hab1pp2ca*, and *ABI1-YFP* overexpression lines 12 days after the seedlings were transferred to ½ MS medium with or without 20 μM ABA. Scale bars, 1 cm. (**B-D**) Phosphorylation of endogenous SnRK2s after 30 min treatments with different concentrations of mannitol (0.3 and 0.6 M) in 7-day-old WT (Col-0), *abi1hab1pp2ca*, *ABI1-YFP* overexpression lines (B), *PP2CA-YFP* overexpression lines (C) or *HAB1-YFP* overexpression lines (D). SnRK2 phosphorylation was detected with anti-phospho-S175-SnRK2s and anti-phospho-S158-SnRK2.1 antibodies. Protein loading was detected by an anti-GFP antibody and Ponceau S staining for RBC. (**E**) Interactions between ABI1 and SnRK2 truncations in yeast two-hybrid

(Y2H) assay. Interactions were determined by yeast growth on media lacking Leu, Trp and His (−L−W−H) after inoculation with saturated culture dilutions (10^{-1}, 10^{-2} and 10^{-3}). Combinations of BD with AD-ABI1 or AD with BD-SnRK2s were used as negative controls. (**F**) Interactions between ABI1 and SnRK2 truncations in *N. benthamiana* leaves using BiFC assays based on split-YFP. At4G33920 was used as control. Scale bars, 10 μm.

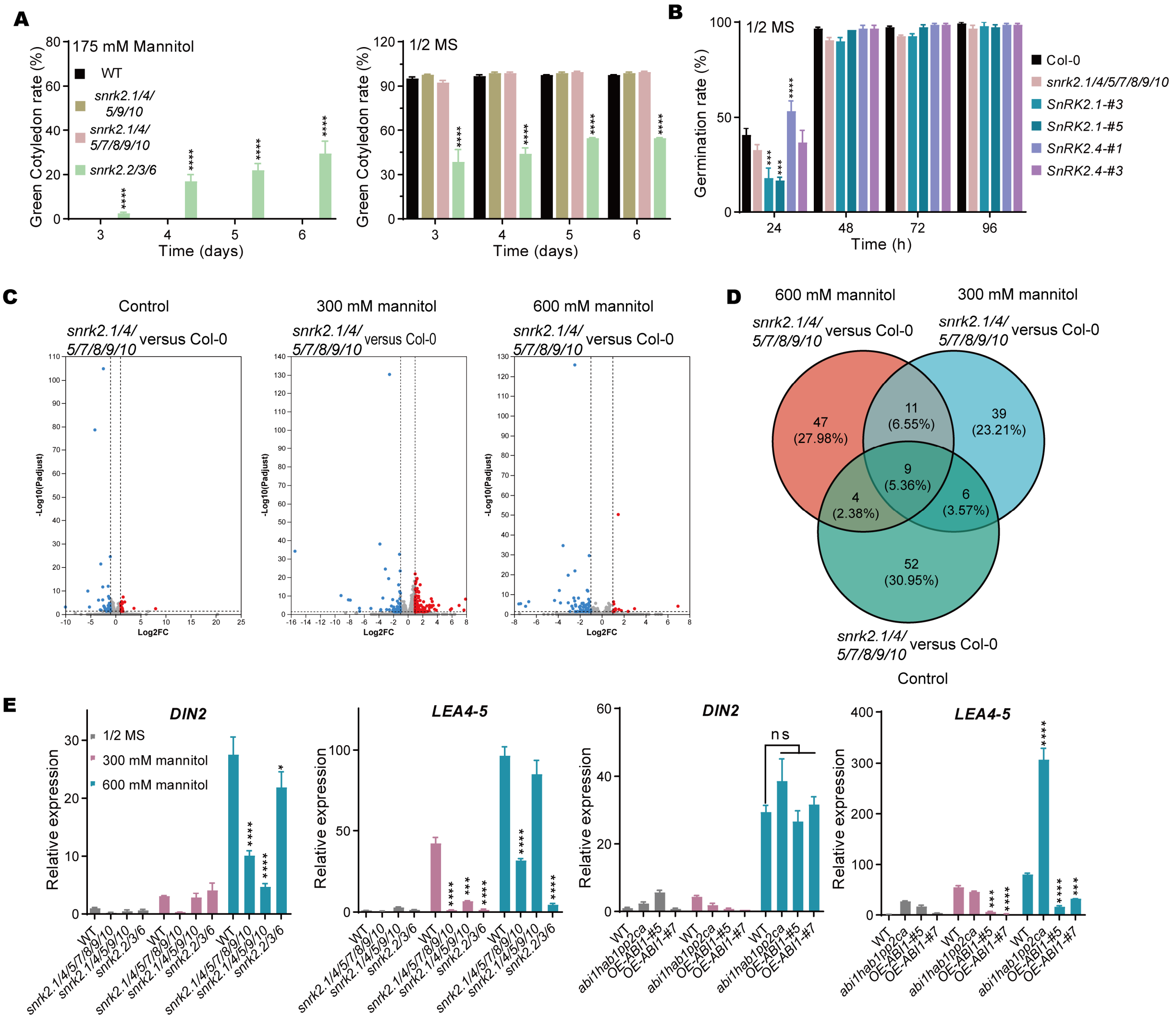

Fig. S6. Subgroup I SnRK2s contribute to osmotic stress resistance.

(**A**) Quantification of the rate of green cotyledons in WT and *snrk2* mutants after sowing on medium supplemented with 175 mM NaCl. (**B**) Seed germination of WT, *snrk2.1/4/5/7/8/9/10* mutant, and *SnRK2pro:SnRK2-GFP* transgenic lines expressing *SnRK2.1-GFP* and *SnRK2.4-GFP* in the *snrk2.1/4/5/7/8/9/10* mutant background, on ½ MS medium. The seed germination rate was quantified. Error bars represent SEM (n = 3 biological independent replicates). (**C**) Volcano plots of some DEGs in the *snrk2.1/4/5/7/8/9/10* mutant compared to wild-type Col-0 under control or mannitol treatment as determined by RNA sequencing. Nine-day-old seedlings were transferred into liquid ½ MS medium with or without 300 mM or 600 mM mannitol and collected for RNA extraction after 6 h of treatment. Genes exhibiting at least two-fold expression changes and an adjusted *P*-value less than 0.01 were deemed upregulated or downregulated. (**D**) Venn diagram showing the overlap of DEGs shown in (B). (**E**) Relative expression of selected osmotic stress-responsive genes in WT, *snrk2* mutants, *abi1hab1pp2ca* mutant, and *ABI1-YFP* overexpression lines, as determined by quantitative reverse transcription PCR (quantitative RT–PCR). Asterisks indicate significant differences (A, B, E), two-way ANOVA followed by a

Tukey test (* $P < 0.05$, ** $P < 0.01$, *** $P < 0.001$, **** $P < 0.0001$). Error bars represent SEM (n = 3 biological independent replicates).

Supplementary Movie 1. **SnRK2.1-GFP droplets fuse to form larger droplets.**

Supplementary Data 1. List of primers used in this study.

Supplementary Data 2. RNA-seq data of WT and the *snrk2.1/4/5/7/8/9/10* mutant following treatments with 300 mM and 600 mM mannitol.
